# Second near-infrared photothermal-amplified immunotherapy using photoactivatable composite nanostimulators

**DOI:** 10.1186/s12951-021-01197-5

**Published:** 2021-12-20

**Authors:** Haitao Sun, Tianzhu Yu, Xin Li, Yangyang Lei, Jianke Li, Xiuhui Wang, Peike Peng, Dalong Ni, Xiaolin Wang, Yu Luo

**Affiliations:** 1 Department of Interventional Radiology, Zhongshan Hospital, Fudan University, Shanghai Institute of Medical Imaging, No. 180 Fenglin Road, Xuhui District, Shanghai, 200032 China; 2 Shanghai Engineering Technology Research Center for Pharmaceutical Intelligent Equipment, Shanghai Frontiers Science Research Center for Druggability of Cardiovascular noncoding RNA, Institute for Frontier Medical Technology, School of Chemistry and Chemical Engineering, Shanghai University of Engineering Science, No. 333 Longteng Road, Shanghai, 201620 People’s Republic of China; 3 Institute for Technical and Macromolecular Chemistry, RWTH Aachen University, Worringerweg 2, 52074 Aachen, Germany; 4 Institute of Translational Medicine, Shanghai University, Shanghai, 200011 People’s Republic of China; 5 School of Basic Medical Sciences, Shanghai University of Traditional Chinese Medicine, Shanghai, People’s Republic of China; 6 Department of Orthopaedics, Shanghai Key Laboratory for Prevention and Treatment of Bone and Joint Diseases, Shanghai Institute of Traumatology and Orthopaedics, Ruijin Hospital, Shanghai Jiao Tong University School of Medicine, 197 Ruijin 2nd Road, Shanghai, 200025 People’s Republic of China

**Keywords:** Second near-infrared light, Nanostimulators, Precise controlled release, Cancer immunotherapy, Photothermal therapy

## Abstract

**Background:**

The construction of a nanoimmune controlled-release system that spatiotemporally recognizes tumor lesions and stimulates the immune system response step by step is one of the most potent cancer treatment strategies for improving the sensitivity of immunotherapy response.

**Results:**

Here, a composite nanostimulator (CNS) was constructed for the release of second near-infrared (NIR-II) photothermal-mediated immune agents, thereby achieving spatiotemporally controllable photothermal-synergized immunotherapy. CNS nanoparticles comprise thermosensitive liposomes as an outer shell and are internally loaded with a NIR-II photothermal agent, copper sulfide (CuS), toll-like receptor-9 (TLR-9) agonist, cytosine-phospho-guanine oligodeoxynucleotides, and programmed death-ligand 1 (PD-L1) inhibitors (JQ1). Following NIR-II photoirradiation, CuS enabled the rapid elevation of localized temperature, achieving tumor ablation and induction of immunogenic cell death (ICD) as well as disruption of the lipid shell, enabling the precise release of two immune-therapeutical drugs in the tumor region. Combining ICD, TLR-9 stimulation, and inhibited expression of PD-L1 allows the subsequent enhancement of dendritic cell maturation and increases infiltration of cytotoxic T lymphocytes, facilitating regional antitumor immune responses.

**Conclusion:**

CNS nanoparticle-mediated photothermal-synergized immunotherapy efficiently suppressed the growth of primary and distant tumors in two mouse models and prevented pulmonary metastasis. This study thus provides a novel sight into photo-controllably safe and efficient immunotherapy.

**Graphical Abstract:**

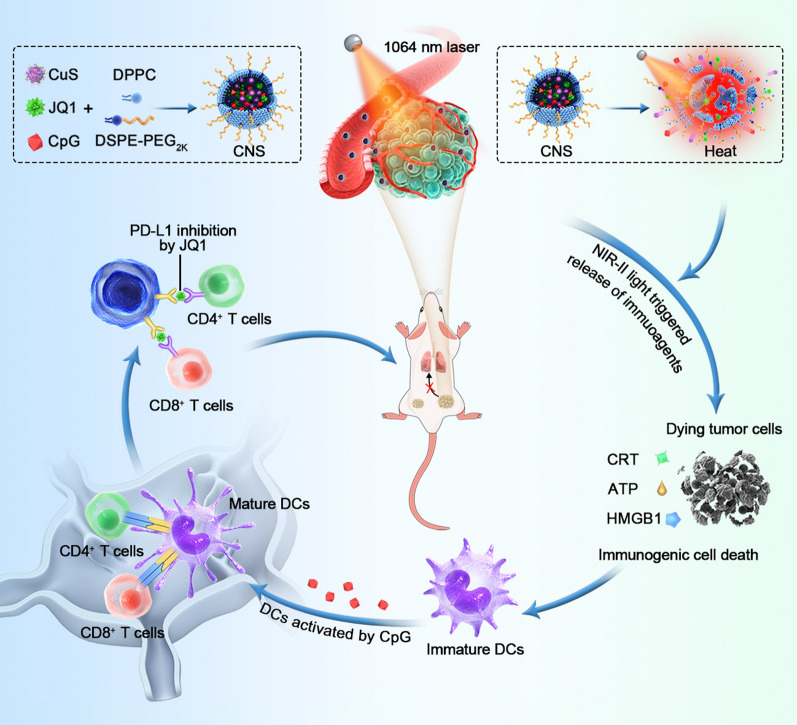

**Supplementary Information:**

The online version contains supplementary material available at 10.1186/s12951-021-01197-5.

## Background

Tumor immunotherapy that mobilizes immune cells to fight malignant tumors is a promising therapeutic approach [1, 2]. Diverse immunotherapy strategies, such as the modulation of immune checkpoint inhibitors (ICBs) against programmed death/ligand 1 (PD-1/PD-L1) [3, 4], cytotoxic T lymphocyte-associated protein 4 (CTLA-4) [5, 6], and adoptive T cell transfer [7–9], have shown satisfactory prospects in clinical studies. Several immune drugs, such as PD-L1 inhibitors and CTLA-4 inhibitors, have been approved by the U.S. Food and Drug Administration (FDA) and successfully commercialized [10]. However, immunotherapy for clinical application has only benefited a small proportion of specific tumor patients due to a low immune response [1, 11]. For instance, tumor patients with an abnormal immune status rarely benefit from checkpoint blockade treatment [12]. Moreover, patients with solid tumors cannot easily be treated using chimeric antigen receptor-redirected T lymphocytes, which only benefit some patients with hematological cancers [13–15]. Other major limiting factors of tumor immunotherapy include serious immune-related systemic toxicity caused by the action of immune drugs in normal tissues and the low efficiency of immune activation in solid tumors [16]. Therefore, the use of clinical immunotherapy with higher efficiency and safety is necessary.

Nanomedicines combining nanotechnology and immunotherapy have shown the potential to improve the efficacy of immunotherapy and minimize toxic side effects [17, 18]. The therapeutic efficiency of nanomedicine-mediated immunotherapy can be strengthened by optimization of pharmacokinetics and toxicity of traditional immune drugs and improving the intratumoral concentration of drugs [19, 20]. Additionally, immunotherapeutic nanomedicines remolded with nanoengineering technology can also improve the efficiency of immune activation via several other mechanisms, such as controlling the timing of immune drug release and/or activation [21], actively targeting immune cells [22, 23], and optimizing the efficiency of drug internalization [24]. These nanomedicines can be designed to respond to external energy fields, such as light [25], ultrasound [26], and magnetic fields [27], thereby achieving the high spatial-temporally release of immune drugs in specific regions. Li et al*.* recently developed an activatable engineered immune device, which can be remotely controlled by near-infrared (NIR) light. In this scenario, NIR light excitation was used to break the photoresponsive linker, releasing active CpG oligonucleotides (ODNs) and precisely activating local intratumoral inflammation without obvious systemic side effects [28]. Therefore, steerable nanomedicines that precisely and safely control cancer immunotherapy are highly promising.

Photothermal therapy (PTT) can act based on the host–guest effect between the laser and material in addition to its familiar high-temporal resolution regulation of drug release and photothermal ablation of tumors [29–31]. The nanomedicine-mediated photothermal effect can ablate tumor cell release of tumor antigens, damage-associated molecular patterns (DAMPs), and immunostimulatory elements, which enable the maturation of dendritic cells (DCs) and activation of immune cells [32–34]. In addition to synergistic effects with immunotherapy, the photothermal effect generated by PTT can also be used to trigger the release of immune agents from nanocarriers by melting the temperature-responsive chemical bonds or nanocarrier structures [35]. Thus, PTT-synergized immunotherapy has great potential to achieve more accurate, effective, and safe immune activation against tumor growth. Sun et al*.* recently constructed an injectable lipid gel encapsulating a photothermal agent (IR820) and anti-PD-L1 antibody (and-L1), which enabled the controllable release of PD-L1 in specific areas, achieving photothermal-assisted immunotherapy [36]. To date, the combined use of PTT with immunotherapy has mostly been based on the excitation of light in the first NIR window (NIR-I, 700–1000 nm), which has inherent limitations of relatively low tissue penetration depth (≤ 1 cm) [37]. Comparatively, in terms of light penetration, the light in the second NIR window (NIR-II, 1000–1700 nm) was elevated three to five-fold (3–5 cm) [38–40]. Phototoxicity analysis showed that the maximum permissible exposure (MPE) of NIR-II light for skin was improved compared with NIR-I light. For example, the MPE of 1064 nm NIR-II light (1 W cm^−2^) was around three-fold higher than that of 808 nm NIR-I light (0.33 cm^−2^) [41]. Therefore, NIR-II PTT-synergized immunotherapy may yield better immune-activating effects in the deeper tissues of patients with solid tumors [42]. However, few studies have investigated NIR-II PTT-activated immunotherapy.

**Scheme 1:**
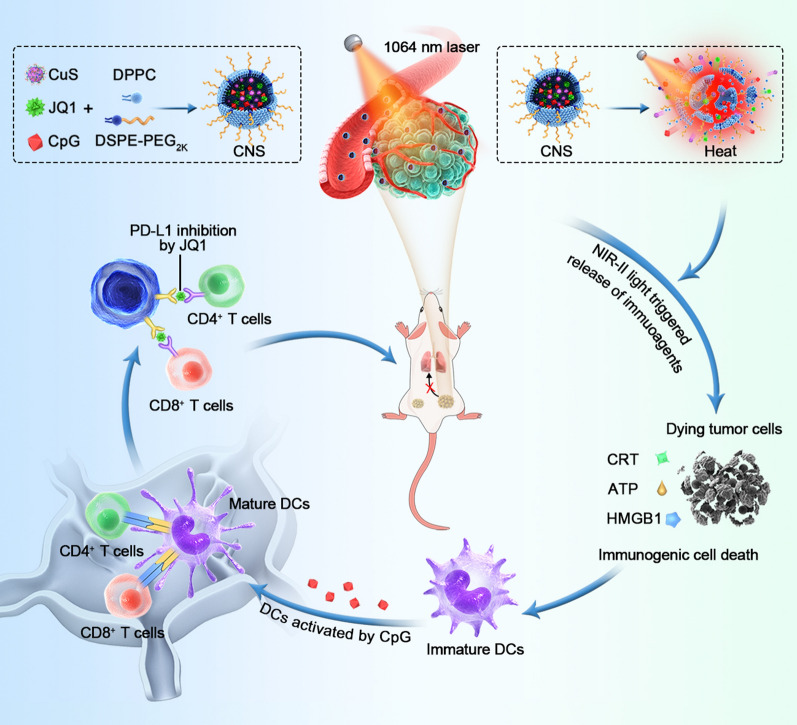
Schematic illustration of NIR-II photoactivated CNSs for remotely controlling photothermal immunotherapy. The simplified synthesis of NIR-II photoactivated CNS and the immune activation cascade of CNS-mediated remotely controlled photothermal immunotherapy are shown

In the present study, different types of novel NIR-photoactivated composite nanostimulator (CNS) were constructed for NIR-II photothermal-synergized immunotherapy. The CNS nanoparticles internally contained copper sulfide (CuS), a NIR-II photothermal conversion agent, two types of immune agent, CpG ODNs, a toll-like receptor-9 (TLR-9) agonist, and JQ1, a PD-L1 inhibitor, which was externally encapsulated by a temperature-sensitive lipid shell (Scheme 1). CuS nanoparticles have recently been considered to be excellent NIR-II photothermal conversion agents due to their obvious absorbance in the NIR-II window, high stability, and good biocompatibility, and have been applied to tumor treatment. The temperature-sensitive lipid shell consisted of thermally sensitive 1,2-dipalmitoyl-*sn-*glycerol-3-phosphocholine (DPPC) and 1,2-distearoyl-*sn-*glycero-3-phosphoethanolamine-poly(ethylene glycol)2000 (DSPE–PEG_2k_), and could be melted at a relatively high temperature (> 41 °C, the phase transition temperature of DPPC), thereby releasing loaded immune drugs into a specific region [43, 44]. CpG, which is a well-known immunoadjuvant studied in phase I–III trials, can facilitate the activation of DCs and enhance cytokine secretion by antigen-presenting cells (APCs) [45, 46]. JQ1, which is a bromodomain and extra terminal protein BRD4 inhibitor, was used to overcome immunological tolerance by downregulating the intratumoral expression of PD-L1 [47]. However, the clearance properties of CpG and potential drug toxicity and water insolubility of JQ1 are limited in their applications for tumor treatment. Thus, the present study used two immune drugs encapsulated in thermally-sensitive liposomes, which were intravenously administered for delivery into the tumor site without being prematurely cleared or released elsewhere. NIR-II photoexcitation of the tumor site enabled CNS-mediated NIR-II PTT to elevate the intratumoral temperature, leading to tumor ablation as well as ICD of tumor cells and releasing of immune-related molecules. Notably, PTT-mediated hyperthermia was able to disrupt the thermally-sensitive lipid layer for the spatiotemporally controllable release of immune agents in the tumor region. Combining ICD and stimulation of TLR-9 are potent promotors of DC maturation in tumor-draining lymph nodes, resulting in an adaptive antitumor immune response. JQ1 can further enhance the intratumoral infiltration of cytotoxic T lymphocytes via downregulation of PD-L1 expression to achieve a more robust antitumor immune activation. CNS nanoparticle-mediated NIR-II photothermal-synergized immunotherapy not only efficiently suppressed the growth of both primary and distant tumors but also greatly inhibited pulmonary metastasis in a murine breast cancer model.

## Material and methods

### Synthesis of BSA–CuS nanoparticles

The synthetic process was briefly divided into three steps. First, CuCl_2_ (0.1 M) dispersed in 2 mL of ultrapure water was added dropwise into BSA (250 mg) dispersed in 50 mL of ultrapure water and reacted for 3 min. Then, NaOH (2 M) was rapidly infused into the mixed solution and stirred for 3 min. Finally, Na_2_S (0.5 M) dispersed in 0.8 mL ultrapure water was rapidly added to the mixed solution. The solution was mixed using magnetic stirring for 3 h. The obtained BSA-CuS solution was purified by centrifugation at 12,000 rpm for 5 min. The supernatant was then dialyzed against water using a dialysis bag (molecular weight cutoff of 8–14 kDa). The purified BSA-CuS nanoparticles (referred to as CuS) were then stored at 4 °C before use.

### Synthesis of photoactivated CNS

DSPE-PEG_2k_, DPPC, and JQ1 with a mass ratio of 5:25:1 were co-dissolved in 10 mL chloroform. The mixture solution was then evaporated to form a thin film using a rotary evaporator. Next, 20 mL of ultrapure water containing CuS (2 mg) and CpG (2 OD) was added into the thin film and stirred at 55 °C for 1 h. After the hydration process, the solution was sonicated by the ultrasonic cell pulverizer in ice bath conditions for 60 min. The obtained solutions were then filtered using a 0.22-μm PVDF syringe-driven filter (Millipore, Bedford, USA) and then purified using ultrafiltration (cutoff molecular weight of 50,000 Da, 5000 rpm) three times to remove unloaded drugs. The obtained CNS_D_ (CuS-CpG-JQ1@lipsome) was stored at 4 °C before use. CNS_0_ (CuS@lipsome), CNS_C_ (CuS-CpG@lipsome), and CNS_J_ (CuS-JQ1@lipsome) were synthesized using the same method. Additionally, ICG-loaded liposomes were constructed for cellular uptake assays in vitro and NIR fluorescence imaging in vivo. ICG@CNS_D_ was synthesized similarly using the hydration-sonication procedure. In brief, DSPE-PEG_2k_, DPPC, and JQ1 with a mass ratio of 5:25:1 were co-dissolved in chloroform. A thin film was obtained through a rotary evaporator. Then, 20 mL ultrapure water containing ICG (2 mg), CuS (2 mg), and CpG (2 OD) and was added into the above thin film, and stirred at 55 °C for 1 h. The subsequent synthesis method was the same as those of CNS_D_. Similarly, ICG@CNS_0_, ICG@CNS_C_, and ICG@CNS_J_ were also obtained through the same method.

The CNS nanoparticle morphologies were imaged using JEM 2100F transmission electron microscope. The DLS and zeta potential of the obtained CNS nanoparticles were analyzed using the Zetasizer Nano series. UV–vis spectrophotometry and HPLC were performed to investigate the loading capacity (LC) and encapsulation efficiency (EE) of CuS, CpG (Cy5.5-CpG, absorbance peak = 650 nm) and JQ1 (λ_abs_ = 260 nm, elution ~ 10 min) of CNS_D_. The colloidal stability of CNS_D_ nanoparticles was evaluated using the Zetasizer Nano series. CNS_D_ dissolved in 1 × PBS was continuously observed for 10 days, and the corresponding DLS and PDI of the nanoparticles were recorded every two days.

### Cellular uptake of CNS nanoparticles

ICG-loaded CNS nanoparticles were used to study the uptake of nanoparticles in both Panc02 and 4T1 cells. First, the Panc02 cells were seeded in 12-well plates at a density of 2 × 10^5^ per well overnight and then co-cultured with ICG@CNS_0_, ICG@CNS_C_, ICG@CNS_J_, and ICG@CNS_D_ for 24 h (20 μg mL^−1^ for all). Cells were then washed three times with PBS and collected to measure the intracellular fluorescence intensity of ICG via flow cytometry. Then, the uptake of nanoparticles in 4T1 cells was conducted and analyzed as described above.

### Cytotoxicity assays and evaluation of therapeutic efficacy in vivo

The safety performance of the nanoparticles was evaluated in vivo. Panc02 cells were plated in 96-well plates and cultured overnight and then co-cultured with CNS_0_, CNS_C_, CNS_J_, and CNS_D_ at various CuS concentrations (0, 12.5, 25, 50, and 100 μg mL^−1^) for 24 h. The cells were then washed three times with PBS and the absorbance of each well was measured at 450 nm using a microplate reader to examine cell viability. The viabilities of various CNS nanoparticles and laser-treated cells were subsequently verified using CCK-8 assays. Panc02 cells were incubated in 96-well plates overnight and then co-cultured with CNS_0_, CNS_C_, CNS_J_, and CNS_D_ at various CuS concentrations (0, 12.5, 25, 50, and 100 μg mL^−1^) for 24 h. The cells were then rinsed three times with PBS and a new medium was added. Each well was irradiated using a 1064 nm laser (1 W cm^−2^) for 5 min and then cultured for 24 h. Finally, the absorbance of each well was measured at 450 nm using a microplate reader.

### In vivo tumor accumulation of CNS nanoparticles

Panc02 tumor-bearing C57BL/6 mice (n = 3) were systemically injected with 0.2 mL of 300 μg/mL CNS@ICG via tail-vein injection. The mice were imaged at different post-injection time points (0, 8, 24, and 36 h) to determine the accumulation of CNS nanoparticles in the tumor. The tumors were imaged using the IVIS imaging system (excitation and emission at 710 and 790 nm, respectively). The corresponding processing software (Living Image software) was performed to record images and calculate the fluorescence intensity of tumors at different time points.

### In vivo photothermal performance of CNS nanoparticles

Panc02 tumor-bearing C57BL/6 mice were randomly divided into four groups (n = 3). The mice in each group were administered 300 μg/mL of CNS_0_, CNS_C_, CNS_J_, and CNS_D_ via tail-vein injection (0.2 mL). Each primary tumor was then photoirradiation using a 1064 nm laser (1 W cm^−2^) for 5 min at 24 h post-injection. The thermal images and corresponding temperature of tumors were recorded using an infrared thermal camera (FLIR 225 s IR thermal).

### In vivo curative effect assessment on Panc02 tumor models

Panc02 tumor-bearing C57BL/6 mice were randomly divided into six groups: control (PBS); CNS_0_ + L; CNS_C_ + L; CNS_J_ + L; CNS_D_; and CNS_D_ + L (n = 5). The mice in each group were intravenously administered with 0.2 mL PBS or 300 μg/mL CNS_0_, CNS_C_, CNS_J_, and CNS_D_. Then, each primary tumor was photoirradiation using a 1064-nm laser (1 W cm^−2^) for 5 min at 24 h post-injection. After the different treatments, the treatment efficacies were assessed by calculating the volumes of the primary and distant tumors every two days for 14 days. Meanwhile, the body weights of Panc02 tumor-bearing C57BL/6 mice were also recorded every two days for 14 days. Tumor volume (V) was calculated using the formula: V = length × width^2^/2, and the relative tumor volumes were calculated as V/V_0_ (where V_0_ represents the original tumor volumes). On day 1 post-treatment, the primary tumors were examined for H&E, TUNEL, and Ki-67 staining to evaluate the intratumoral treatment effect. On day 14 post-treatment, the mice in each group were euthanized and the corresponding tumor weights of both the primary and distant tumors were calculated.

### In vivo treatment and antimetastatic efficacy evaluation on 4T1 tumor models

4T1 tumor-bearing Balb/c mice were randomly divided into six groups: control (PBS); CNS_0_ + L; CNS_C_ + L; CNS_J_ + L; CNS_D_; and CNS_D_ + L (n = 5). The mice in each group were intravenously administered with 0.2 mL of PBS or 300 μg/mL CNS_0_, CNS_C_, CNS_J_, and CNS_D_. Each primary tumor was then photoirradiation using a 1064 nm laser (1 W cm^−2^) for 5 min at 24 h postinjection. After the different treatments, the treatment efficacies were assessed by calculating the volumes of both primary and distant tumors every two days for 14 days. Meanwhile, the body weights of 4T1 tumor-bearing Balb/c mice were also recorded every two days for 14 days. Tumor volume (V) was calculated using the formula: V = length × width^2^/2, and the relative tumor volumes were calculated as V/V_0_ (where V_0_ represents the original tumor volumes). On day 1 post-treatment, the tumors were examined for H&E staining to evaluate the intratumoral treatment effects.

The mice in the different treatment groups (n = 3) were euthanized to collect lung tissue at 30 days post-treatment to study the antitumor lung metastasis of CNS nanoparticles. The metastatic nodules in the lungs were photographed and H&E staining was performed.

### In vivo biocompatibility assessment of CNSD

Panc02 tumor-bearing C57BL/6 mice in each group were sacrificed at the end of treatment. Major organs including the heart, liver, spleen, lung, and kidney were collected and examined by H&E staining. The long-term biocompatibility of CNS_D_ was also assessed in healthy female C57BL/6 mice. The mice in the treated groups were intravenously injected with 200 μL of 300 μg/mL CNS_D_. At day 0 and various post-injection time points (days 15 and 30), blood samples were examined for routine blood and biochemical analysis (n = 3). The corresponding major organs (heart, liver, spleen, lung, and kidney) were also examined by H&E staining at 0, 15, and 30 days.

### Statistical analysis

Data are shown as mean ± standard deviation. One-way analysis of variance (ANOVA) with Tukey’s multiple comparisons test was performed to analyze the statistical significance among different groups. Statistical significance was divided as three categories: *p < 0.05 **p < 0.01, and ***p < 0.001.

## Results and discussion

### Synthesis and characterization of CNS

Various photoactivated CNS loading agents were prepared using a film hydration method [48]. In brief, a thin film composed of DPPC (MW = 734.0) and DSPE–PEG_2k_ (MW = 2805.5) with a feeding mass ratio of 25:5 was synthesized and then hydrated with ultrapure water including CuS, JQ1, and CpG. Hydrophobic JQ1 and hydrophilic CuS and CpG were encapsulated into temperature-responsive liposomes through hydrophobic and π–π stacking interactions. The EE of CNS_D_ was 71.8% for CuS, 57.2% for JQ1, and 69.8% for CpG, and the corresponding LC values were 4.3%, 1.7%, and 0.1%, respectively. Three control counterparts (CNS_0_, CNS_C_, and CNS_J_) were simultaneously prepared using a similar process and used as a comparison.

**Fig. 1:**
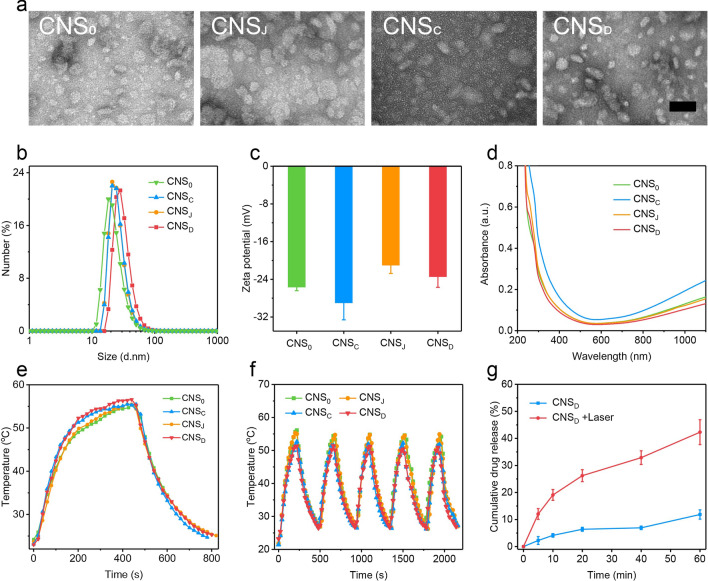
**a** Typical TEM images of CNS_0_, CNS_J_, CNS_C,_ and CNS_D_, the scale bar = 50 nm. **b** Typical DLS profiles of CNS_0_, CNS_J_, CNS_C_, and CNS_D_. **c** Zeta potentials of CNS_0_, CNS_J_, CNS_C,_ and CNS_D_. **d** UV–vis absorption spectra of CNS_0_, CNS_J_, CNS_C_, and CNS_D_. **e** Photothermal effect of 100 µg/mL CNS_0_, CNS_J_, CNS_C_, and CNS_D_ with 1064-nm irradiation (1 W cm^−2^) followed by laser shut-off. **f** Photothermal stability assay of CNS_0_, CNS_J_, CNS_C_, and CNS_D_ after five cycles of laser on/off. **g** Release of JQ1 from CNS_D_ with or without laser irradiation (1064-nm, 1 W cm^−2^) for 5 min (the concentrations of CuS and JQ1 for CNS_D_ were 100 and 39.5 µg/mL, respectively)

As shown in the transmission electron microscopy (TEM) images, CNS_D_ and the three control counterparts (CNS_0_, CNS_C_, and CNS_J_) were quasi-spherical and showed a uniform size distribution (Fig. 1a). Dynamic light scattering (DLS) revealed that the hydrodynamic size of CNS_D_ was approximately 31.3 ± 1.4 nm, which was slightly larger than that of CNS_0_ (25.1 ± 1.1 nm), CNS_C_ (27.0 ± 2.7 nm), and CNS_J_ (26.2 ± 3.7 nm) (Fig. 1b). Moreover, the polydispersity indexes (PDIs) of CNS_0_, CNS_C_, CNS_J_, and CNS_D_ were 0.23, 0.22, 0.24, and 0.23, respectively, suggesting good mono-dispersity of the four types of nanoparticles. The zeta potential was measured to investigate the surface charge of various prepared nanoparticles. Compared with CNS_C_ (− 28.9 mV), CNS_D_ (− 23.4 mV) in 1 × phosphate-buffered saline (PBS) showed an elevated surface charge, indicating successful JQ1 loading. Compared with CNS_J_ (− 20.9 mV), CNS_D_ (− 23.4 mV) in 1 × PBS showed a reduced surface charge, indicating successful CpG ONDs encapsulation (Fig. 1c). CNS_D_ and the three control counterparts (CNS_0_, CNS_C_, and CNS_J_) showed similar peaks in the ultraviolet–visible (UV–vis) absorption spectrum, indicating the inappreciable affection of loaded two drugs on the optical property of the prepared nanoparticles (Fig. 1d). Furthermore, the prepared CNS_D_ nanoparticles showed excellent colloidal stability due to their stable hydrodynamic size and PDI over 10 days (Additional file 1: Fig. S1).

### Photothermal properties and photothermal-induced drug release performance

The NIR-II photothermal properties and photothermal-induced release of immune agents from CNS were further investigated. Under conditions of photoirradiation (1064 nm) at a power intensity of 1 W cm^−2^, the temperatures of CNS_0_, CNS_C_, CNS_J_, and CNS_D_ solution showed an obvious increase up to around 55 °C in 7 min (Fig. 1e; Additional file 1: Fig. S2). The photothermal temperature curves exhibited minimal differences after five cycles of heating and natural cooling (Fig. 1f), showing good photothermal stability of the nanoparticles. Furthermore, the photothermal conversion efficiency of CNS_D_ was ≈ 32.6%, which was similar to that of CNS_0_ (32.4%), CNS_C_ (31.8%), and CNS_J_ (31.5%) (Additional file 1: Fig. S3).

The photo-triggered release properties of the loaded immune agents were further analyzed. Under conditions of photoirradiation (1064 nm) at a power intensity of 1 W cm^−2^ for 5 min, the cumulative releases of JQ1 and CpG from CNS_D_ were 42.3% and 44.7% in 1 h, respectively, which were 3.6- and 3.5-fold higher than that without laser irradiation (Fig. 1g; Additional file 1: Fig. S4). The underlying mechanism can be explained as follows: CNS_D_ mediated the photothermal effect and induced the dissociation of temperature-responsive liposomes with a phase-transition temperature of around 42 °C, resulting in the on-demand release of drugs.

### CNS-mediated intracellular photothermal-activated immune response

**Fig. 2:**
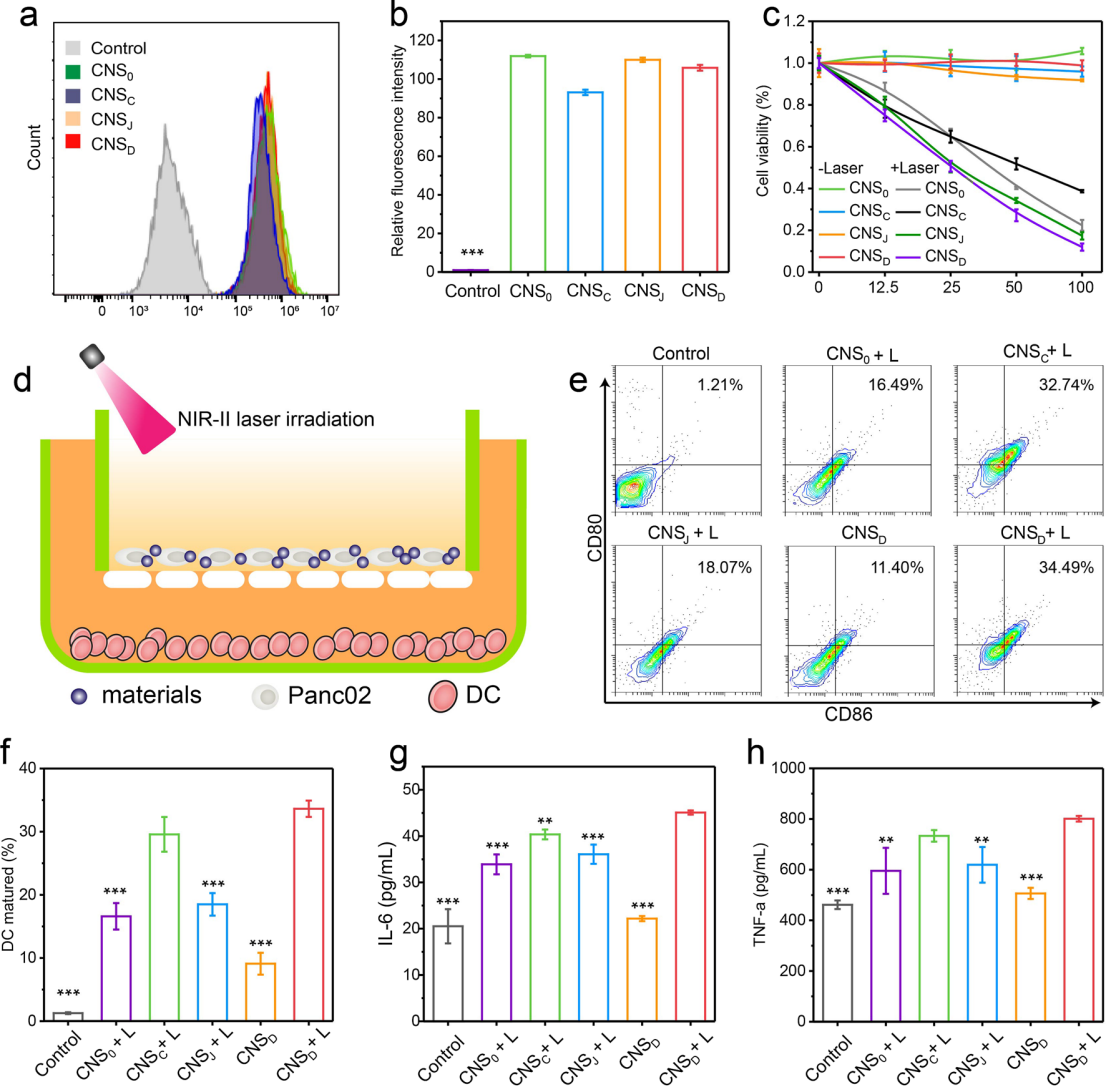
**a** Fluorescence intensity of Panc02 cells treated with PBS (control) or various ICG-loaded CNS nanoparticles ([ICG] = 20 µg mL^−1^) for 24 h via flow cytometry. **b** Corresponding relative ICG fluorescence intensity of Panc02 cells after treatment with PBS (control) or various ICG-loaded CNS nanoparticles ([ICG] = 20 µg mL^−1^) for 24 h via flow cytometry (n = 3). **c** Cell viabilities of Panc02 cells after treatment with various types of CNS at different concentrations (0, 12.5, 25, 50 and 100 μg/mL) with or without 1064-nm laser illumination (1 W cm^−2^) for 5 min. **d** Schematic illustration of CNS-induced DCs maturation in vitro. **e** Flow cytometry assay of mature DCs (CD11c^+^CD80^+^CD86^+^) in the different groups. **f** Corresponding quantitative percentages of mature DCs in different groups (n = 3); **g**, **h** Quantification of secretion levels of IL-6 (**g**) and TNF-α (**h**) in DC suspension (n = 3). **p < 0.01, *** p < 0.001

The cellular uptake of CNS was first analyzed in Panc02 cells. The cellular uptake of CNS was confirmed by loading 2.5% (w/w) CNS with indocyanine green (ICG), which is a cyanine dye approved by the FDA, to construct four loaded CNS nanoparticles (CNS_0_@ICG, CNS_C_@ICG, CNS_J_@ICG, and CNS_D_@ICG) (Additional file 1: Fig. S5). The ICG-loaded CNS nanoparticles exhibited identical peaks in the UV–vis absorption spectrum, indicating their successful preparation (Additional file 1: Fig. S6). After a 24-h incubation, the fluorescence intensity of CNS_D_ cultured in Panc02 cells and 4T1 cells was 105- and 109-fold greater than that of the control group, respectively, suggesting the effective internalization of nanoparticles into cells. There were no obvious differences in fluorescence intensities in both Panc02 cells and 4T1 cells cultured with the four nanoparticles (Fig. 2a, b; Additional file 1: Fig. S7). The cytotoxicity of these CNS nanoparticles was analyzed. The viability of the Panc02 cells was > 90% after incubation with four CNS nanoparticles for 24 h, indicating the nontoxicity and good biocompatibility of CNS in vitro (Fig. 2c). The capacity for CNS to suppress tumor cells was further assessed. After incubation with CNS_0_, CNS_C_, CNS_J,_ or CNS_D_ for 24 h, Panc02 cells were exposed to a 1064 nm NIR laser at a power density of 1 W cm^−2^ for 5 min. The results showed that cells treated with diverse CNS nanoparticles plus NIR-II light all decreased with elevating CuS concentrations (Fig. 2c). Using 100 μg/mL CuS, the viabilities of the Panc02 cells treated with CNS_0_ + L, CNS_C_ + L, CNS_J_ + L, and CNS_D_ + L decreased to 24.6%, 38.7%, 17.3%, and 11.9%, respectively, suggesting excellent CNS-mediated photothermal effects against cell growth.

Exposure of the tumor cells to calreticulin (CRT) as a specific signal for triggering APCs was a characteristic sign of ICD induction. Thus, CRT exposure of the Panc02 cells was further evaluated to validate the performance of CNS-mediated ICD under conditions of NIR-II irradiation in vitro. 24 h after NIR-II laser irradiation (1064 nm, 1.0 W cm^−2^, 5 min), the mean fluorescence intensity (MFI) of CRT in Panc02 cells treated with the four CNS nanoparticles was elevated. Of note, the MFI of CRT in the CNS_D_ + L group was 3.2 and 2.5-fold higher than that in the control and CNS_D_ groups, respectively (Additional file 1: Fig. S8). These data indicate that CNS nanoparticles were capable of efficiently triggering ICD under conditions of NIR-II laser irradiation.

As a key role in human immunity, immature DCs can engulf antigens and transfer them to neighboring lymph nodes, where they are processed into peptides for T cell activation. Mature DCs can be detected by the co-stimulatory molecules, CD80/CD86, which are representative markers that signify DC maturation. Murine bone marrow-derived dendritic cells (BMDCs) extracted from C57 mice received different treatments and the corresponding percentages of matured DCs (CD11c^+^CD80^+^CD86^+^) were then measured by flow cytometry (Fig. 2d). NIR-II laser irradiation significantly elevated the maturation of various CNS-treated DCs compared with the non-laser irradiation group (Fig. 2e). DC maturation in the CNS_D_ + L group was 26.4-fold higher than in the PBS group. Notably, DC maturation in the CNS_D_ + L group was 3.7, 2.0, and 1.8-fold higher than that of the CNS_D_, CNS_0_ + L, and CNS_J_ + L groups, respectively, suggesting that the NIR-mediated photothermal effect can be attributed to the laser-induced release of tumor antigens and loaded immune-agents (Fig. 2f). However, DC maturation was similar in the CNS_D_ + L and CNS_C_ + L groups, which may be explained by the fact that JQ1 does not directly facilitate DC maturation.

Immune-related cytokines, such as interleukin-6 (IL-6) and tumor necrosis factor-α (TNF-α), can be secreted by mature DCs. Thus, we measured the concentration of cytokines in the medium using ELISA kits. The secretion levels of TNF-α and IL-6 in the NIR-II laser-treated CNS groups were all increased (Fig. 2g, h). In particular, levels of IL-6 and TNF-α in the CNS_D_ + L group were 2.0- and 1.6-fold, respectively, higher than that of the CNS_D_ group.

### CNS-mediated synergistic NIR-II photothermal immunotherapy in Panc02 tumors

**Fig. 3:**
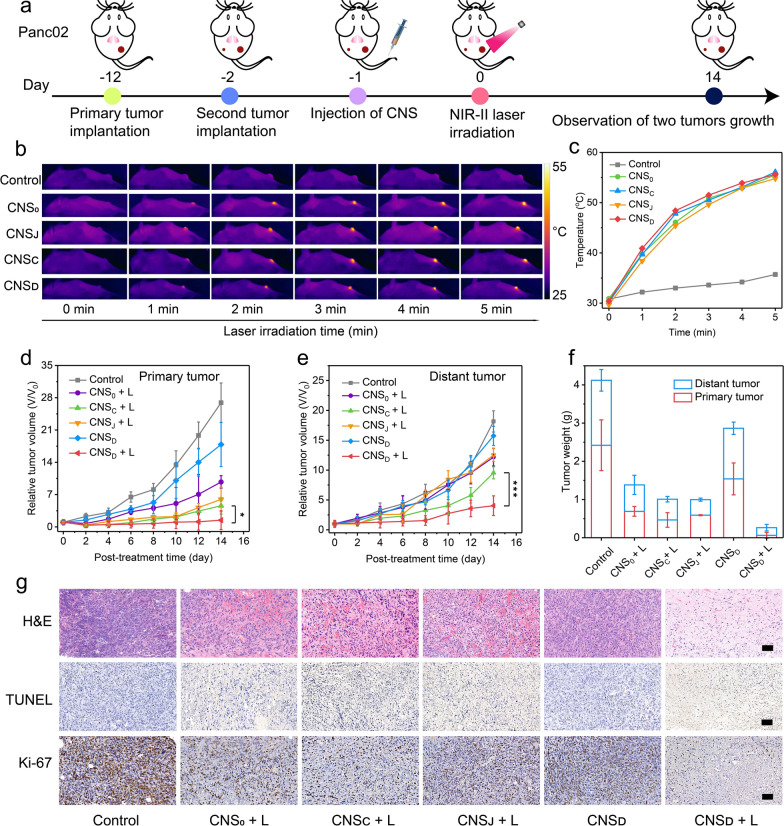
**a** Schematic illustration of Panc02 tumor implantation, treatment, and monitoring. **b** NIR thermal photos of Panc02 tumor-bearing mice under laser irradiation at 24 h post-injection of 200 μL of PBS (control), or 300 µg/mL CNS_0_, CNS_C_, CNS_J_, or CNS_D_ via tail-vein injection. **c** Temperature increase curves in the tumor areas of Panc02 tumor-bearing mice after administration of PBS (control), CNS_0_, CNS_C_, CNS_J_, and CNS_D_ under NIR-II laser illumination (1064 nm, 1 W cm^−2^, 5 min). **d**, **e** Relative tumor volumes of primary mass (**d**) and distant masses (**e**) in Panc02 tumor-bearing C57BL/6 mice (n = 5) intravenously administered with 200 μL of PBS or 300 µg/mL CNS_0_, CNS_C_, CNS_J_, or CNS_D_ in the presence and absence of photoirradiation. **f** The tumor weight of primary masses and distant masses in the Panc02 tumor-bearing C57BL/6 mice (n = 5) in the control, CNS_0_, CNS_C_, CNS_J_, and CNS_D_ groups at the end of treatments. **g** The representative H&E, TUNEL, and Ki-67 immunofluorescence staining images of primary tumors in various groups (the scale bar represents 50 μm). *p < 0.05, ***p < 0.001

Panc02 tumor-bearing C57BL/6 mice with primary and distant tumors were used to evaluate the curative effect of CNS-mediated NIR-II photothermal immunotherapy in living mice. The mice were intravenously administered with various CNS nanoparticles and the primary tumor was then exposed to NIR-II laser treatment. The growth of primary and distant tumors was subsequently monitored for two weeks (Fig. 3a). The ICG labeled CNS nanoparticles were intravenously injected into the Panc02 tumor-bearing mice to investigate the optimal tumor accumulation of CNS. The fluorescence intensity in tumors of CNS_0_, CNS_C_, CNS_J_, and CNS_D_-treated mice gradually increased and peaked at 24 h post-injection (Additional file 1: Fig. S9a). At this time-point, the intensity of the tumors of CNS-injected mice was at least 5.9-fold higher relative to the background, demonstrating the accumulation of nanoparticles into tumors (Additional file 1: Fig. S9b).

The photothermal properties of CNS nanoparticles were further investigated in both Panc02- and 4T1 tumor-bearing mice to examine the effects of PTT in vivo. At 24 h post-treatment using various CNS nanoparticles, the primary tumors of the mice were exposed to the NIR-II laser (1064 nm) at a power intensity of 1 W cm^−2^ for 5 min. The temperatures of the region of Panc02 tumors in all CNS-treated groups were gradually elevated and peaked at least ≈54.8 °C for 5 min post-irradiation (Fig. 3b, c). Similarly, the temperatures of the region of 4T1 tumors in all treated groups were at least ≈54.2 °C for 5 min post-irradiation (Additional file 1: Fig. S10). These results showed that the CNS nanoparticles have good photothermal properties in vivo*.* Furthermore, the tumor temperatures in all treated groups were similar at the same time points, showing that all CNS nanoparticles possessed similar photothermal conversion efficacies and intratumoral aggregation.

Due to the good photothermal conversion efficiency and excellent intratumoral aggregation properties of CNS nanoparticles, Panc02 tumor-bearing mice were then randomly divided into six groups to investigate the antitumor capacities of various CNS nanoparticles. After treatment, the curative effects of CNS in vivo were assessed by monitoring the growth of the primary and distant tumors. In the absence of photoirradiation, the growth of primary and distant tumors in CNS_D_ and PBS-treated mice showed negligible inhibition (Fig. 3d, e). Following photoirradiation, the primary tumor volume in the CNS_D_-treated group was effectively suppressed and was 6.7, 4.1, and 3.2-fold lower than CNS_0_, CNS_C_, and CNS_J_-treated mice, respectively. The distant tumor volume in the CNS_D_-treated group was also effectively inhibited and was 3.0, 2.4, and 3.1-fold lower than CNS_0_, CNS_C_, and CNS_J_-treated mice, respectively. Furthermore, the tumor weights of the primary and distant tumors in the CNS_D_ + L group were 0.06 and 0.2 g, which was 25.7 and 6.6-fold lower than that in the CNS_D_ group, respectively (Fig. 3f). The pathological data from the CNS_D_ + L group exhibited larger regions of cell apoptosis and necrosis [hematoxylin and eosin (H&E) and terminal deoxynucleotidyl transferase dUTP nick end labeling (TUNEL)] in the primary tumors compared with the CNS_0_ + L, CNS_C_ + L, and CNS_J_ + L groups (Fig. 3g). The PBS and CNS_D_ groups showed no obvious areas of apoptosis in these masses. Similarly, Ki-67 staining images showed the greatest inhibition of cancer cell proliferation in the CNS_D_ + L group.

### CNS-mediated synergistic NIR-II photothermal immunotherapy on 4T1 tumors

**Fig. 4:**
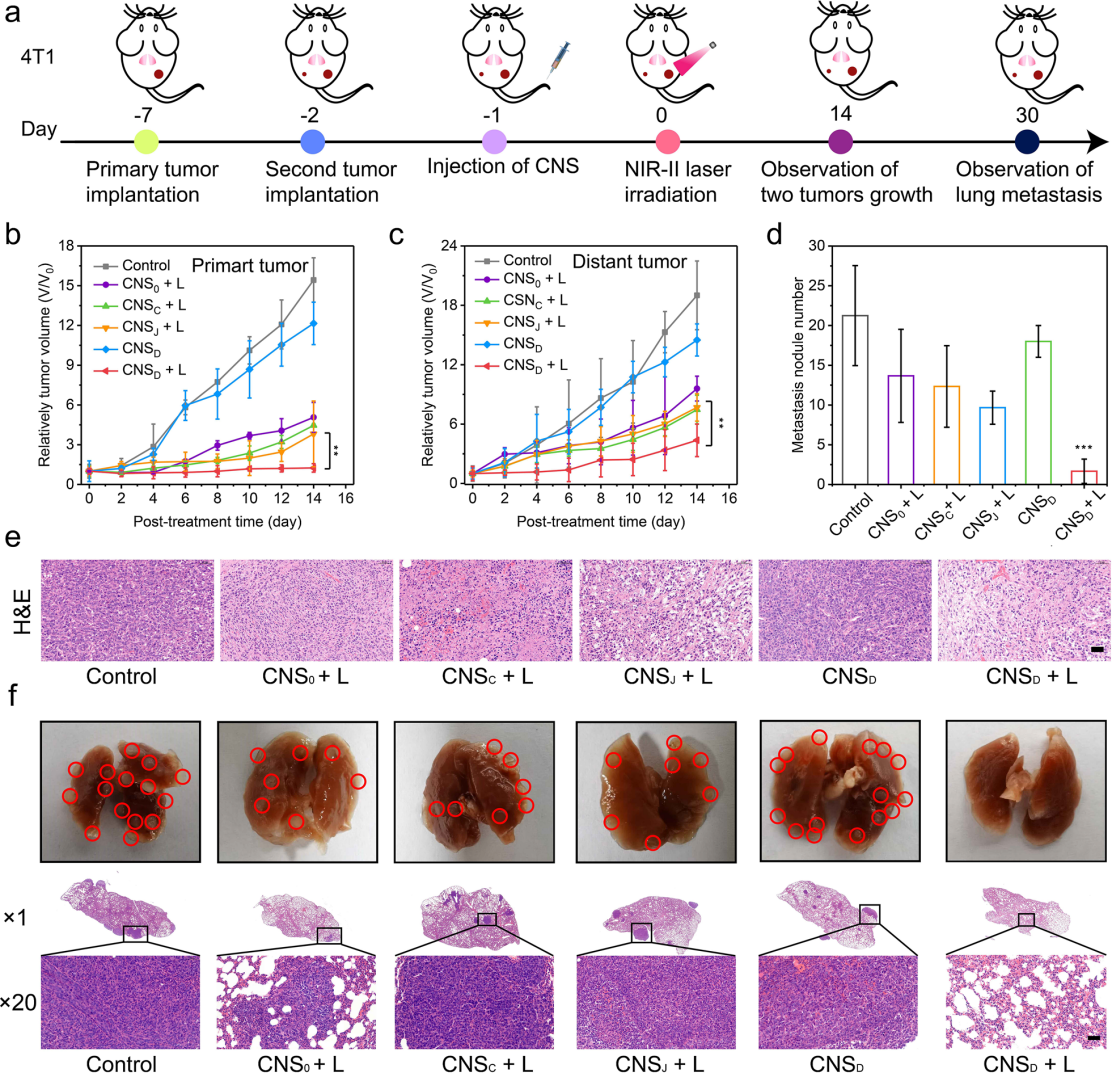
**a** Schematic illustration of 4T1 tumor implantation, treatment, and monitoring. **b**, **c** Relative tumor volumes of primary masses (**b**) and distant masses (**c**) in 4T1 tumor-bearing Balb/c mice (n = 5) intravenously administered with 0.2 mL of PBS (control) or 300 µg/mL CNS_0_, CNS_C_, CNS_J,_ or CNS_D_. **d** Metastatic nodules of the lung in the various groups. **e** H&E staining of the 4T1 tumors at day 1 after various treatments (the scale bar represents 50 μm). **f** Digital photos of murine lung tissues and the corresponding H&E staining of the lung tissues at the end of treatments (n = 3, the scale bar represents 50 μm). **p < 0.01 and ***p < 0.001

4T1 tumor-bearing Balb/c mice with primary and distant tumors were given various treatments and monitored to investigate the efficacy of CNS nanoparticles-mediated photothermal immunotherapy in inhibiting lung metastasis (Fig. 4a). The inhibition effects of both primary and distant tumors were investigated due to the desirable photothermal effects of CNS nanoparticles in 4T1 tumor-bearing mice. In contrast to the results of the Panc02 tumor-bearing mouse model, the growth of primary and distant tumors was not suppressed in the control and CNS_D_ groups. However, photoirradiation showed the greatest suppressive effects of both primary and distant tumors in CNS_D_-treated mice compared with CNS_0_, CNS_C_, and CNS_J_-treated mice (Fig. 4b, c). Compared with the other groups, the CNS_D_ + L group exhibited the most apoptosis of tumor cells, as demonstrated by the H&E staining of the tumor biopsy (Fig. 4e). The lungs of the 4T1 tumor-bearing mice were extracted and pathologically examined 30 days after various treatments to investigate the therapeutic effects of CNS-mediated inhibition of lung metastasis (Fig. 4d, f). The greatest inhibitory effect of tumor lung metastasis was observed in CNS_D_-treated mice. Furthermore, the average number of pulmonary metastasis in the CNS_D_ + L group was at least six times lower than that in the other groups. These data showed that CNS_D_ nanoparticles possessed the potential to inhibit diverse malignant tumors and prevent tumor metastasis.

### CNS-mediated synergistic immune response in vivo

The mechanisms of CNS-mediated photothermal immune response in vivo were further evaluated due to the superior suppression of primary tumors and metastatic tumors.

**Fig. 5:**
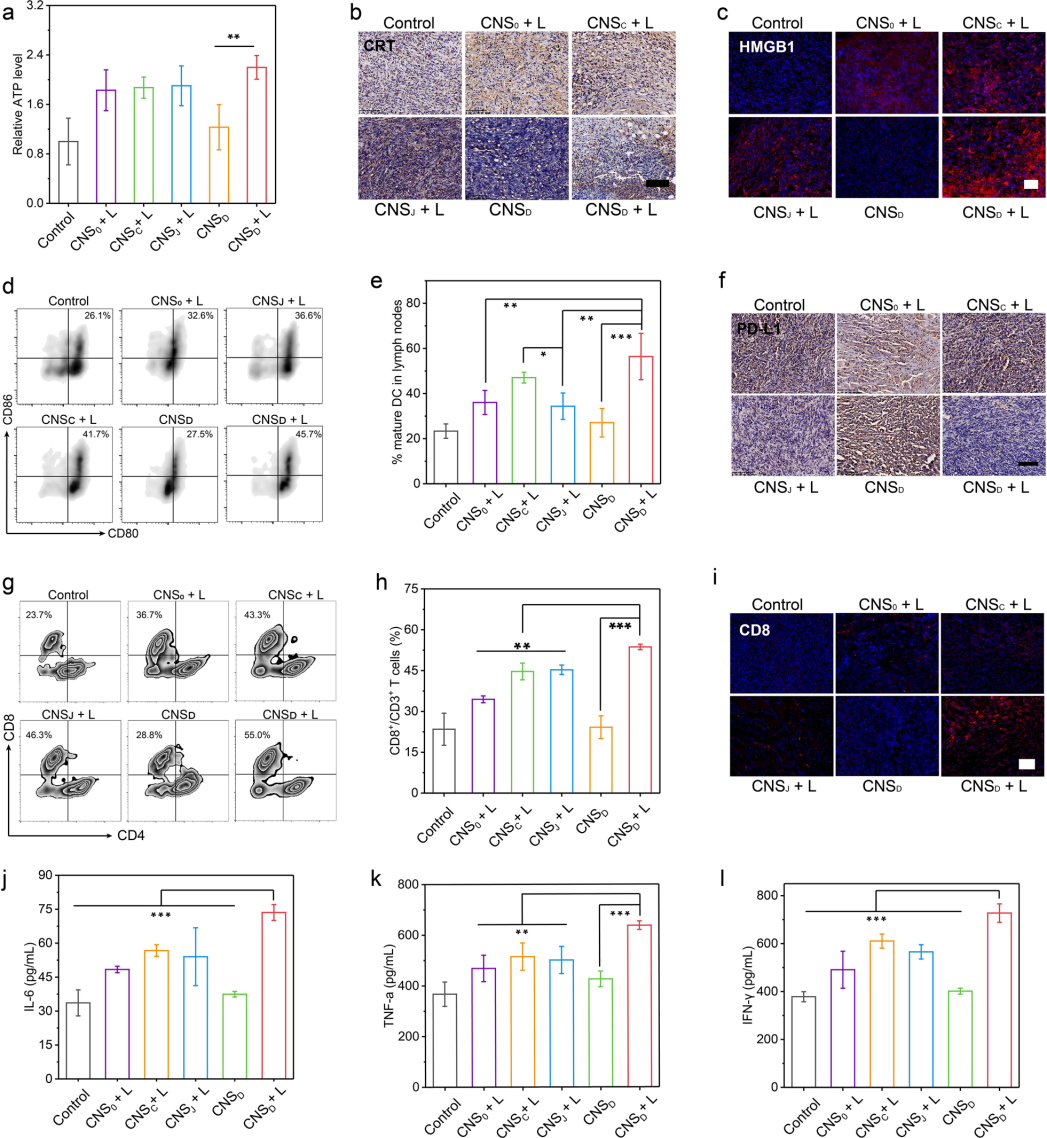
NIR-II photoactivated immune activation in vivo. **a** Intratumoral levels of ATP in Panc02tumor-bearing C57BL/6 mice in the different groups after intravenous injection of 200 µL of PBS or 300 µg mL^−1^ CNS_0_, CNS_C_, CNS_J,_ or CNS_D_. **b** Immunohistochemical staining photos of CRT in tumors sections with different treatments at day 1 post-treatment (blue signal represents cell nuclei and brown signal represents CRT; the scale bar represents 100 μm). **c** Immunofluorescence staining photos of HMGB1 in tumor sections with different treatments at day 1 post-treatment (blue signal represents cell nuclei and brown signal represents HMGB1; the scale bar represents 50 μm). **d**, **e** Flow cytometric plots (**d**) and corresponding quantitative percentages (**e**) of matured DCs (CD80^+^CD86^+^ gated on CD11c^+^) in tumor-draining lymph nodes of Panc02 tumor-bearing C57BL/6 mice in various groups at day 3 post-treatment. **f** Immunohistochemical staining photos of PD-L1 in tumors sections with different treatments at day 14 post-treatment (blue signal represents cell nuclei and brown signal represents PD-L1; the scale bar represents 100 μm). **g**, **h** Flow cytometric plots (**g**) and corresponding quantitative percentages (**h**) of CD3^+^/CD4^+^ T cells and CD3^+^/CD8^+^ T cells in distant tumors of Panc02 tumor-bearing C57BL/6 mice in various groups at day 10 post-treatment. **i** Immunofluorescence staining of CD8 in tumors sections with different treatments at day10 post-treatment (blue signal represents cell nuclei and brown signal represents HMGB1; the scale bar represents 50 μm). **j**–**l** Serum levels of cytokines including IL-6 (**j**), TNF-α (**k**), and IFN-γ (**l**) in Panc02 tumor-bearing C57BL/6 mice in various groups at day 3 post-treatment. *p < 0.05, **p < 0.01, and ***p < 0.001

An in-depth analysis of the key processes of the immune response in vivo, including ICD induction, DC maturation, and T cell infiltration, was performed to investigate the CNS-based photothermal immune-activation and the synergic effects. Since the induction of ICD is the first step in photothermal immune activation, activation of ICD triggered by CNS nanoparticles under laser irradiation was analyzed first. The representative biomarkers of ICD including adenosine triphosphate (ATP), CRT, and expression of high mobility group box 1 protein (HMGB1) were used to facilitate the uptake, processing, and presentation of tumor antigens in DCs. One day after treatment, the intratumoral ATP levels of various CNS nanoparticles plus laser irradiation were elevated and compared with the single PBS-treated and CNS_D_-treated groups. The ATP levels of the CNS_D_ + L group were 2.2 and 1.8-fold higher than those of the control and CNS_D_ groups, respectively (Fig. 5a). Moreover, there was a negligible difference in intratumoral ATP levels between the four CNS nanoparticles with photoirradiation, showing that ATP was mainly influenced by NIR-irradiated CNS nanoparticles. Immunohistochemical staining of the tumor section revealed CRT in the tumors as a brown signal. Almost no obvious brown signal was shown in the tumors after treatment with CNS_D_ alone or PBS (Fig. 5b). In contrast, tumors treated with various CNS nanoparticles with photoirradiation exhibited an obvious enhanced brown signal. HMGB1 was shown as a red fluorescent signal in the immunofluorescence staining images. Similarly, compared with the CNS_D_ alone and PBS-treated groups, tumors treated with diverse CNS nanoparticles with photoirradiation exhibited significantly enhanced red fluorescence signals (Fig. 5c). These data suggested that CNS-mediated NIR-II PTT was able to induce intratumoral ICD.

The tumor-draining lymph nodes in C57BL/6 mice were collected after treatment and measured using flow cytometry to evaluate DC maturation (CD11c^+^CD80^+^CD86^+^) (Additional file 1: Fig. S11). The percentage of matured DCs in CNS_D_-treated mice with laser irradiation was the highest, and was 2.4, 2.1, and 1.6-fold higher than that in the control, CNS_D_, and CNS_J_ + L groups, respectively, suggesting that CNS-mediated hyperthermia could activate DCs maturation via the synergic action of PTT-induced ICD and the photothermal-induced release of immune-agents (Fig. 5d, e). As for PD-L1 expression, the inhibitory effect of JQ1 on PD-L1 was firstly be verified in vitro. The western-blot analysis showed that the free or released JQ1 was able to effectively downregulate the IFN-γ-stimulated PD-L1 expression (Additional file 1: Fig. S12). Then, since JQ1 facilitates the downregulation of PD-L1 expression, we examined whether NIR-II laser-induced JQ1 released from CNS_D_ could suppress PD-L1 expression in Panc02 tumor xenografts in vivo by examining immunohistochemical staining of the tumor section. As shown in the representative immunohistochemistry staining images, the brown signal intensity in the CNS_D_ + L and CDS_J_ + L groups was significantly weakened compared with those in the other treatment groups (Fig. 5f). The brown signal intensity in the CNS_D_ group without photoirradiation did not decrease compared with that in the CNS_D_ group with photoirradiation, implying that tumor PD-L1 expression decreased due to NIR laser-induced JQ1 release from CNS_D_ nanoparticles.

Cytotoxic T lymphocytes, such as CD8^+^ T cells, can identify and kill tumor cells [49]. Hence, tumor tissues were collected after various treatments to assess the percentage of cytotoxic T lymphocytes cells (CD8^+^/CD3^+^) via flow cytometric analysis to investigate the CNS-mediated synergized antitumor immune response (Additional file 1: Fig. S13). Levels of CD8^+^ T cells in various CNS nanoparticles-treated mice were all increased after photoirradiation compared with those without photoirradiation. In particular, the percentages of T cells (CD8^+^/CD3^+^) in the CNS_D_ + L group were 1.6, 1.2, and 1.2-fold higher than those in the CNS_0_ + L, CNS_C_ + L, and CNS_J_ + L groups, respectively (Fig. 5g, h). Similarly, immunofluorescence analysis revealed a greater red fluorescence intensity in the CNS_D_ + L group for CD8 than that in other groups, which is in keeping with the flow cytometry results (Fig. 5i). These results indicated that CNS_D_-mediated photothermal immunotherapy enabled the acquisition of synergetic antitumor immune response by combining the photothermally released immunoregulators and photothermal-induced immune response. Furthermore, the secretion of cytokines, such as IL-6, TNF-α, and interferon-gamma (IFN-γ), is a key indicator of an antitumor immune response [21]. Therefore, the levels of these cytokines in the serum of Panc02 tumor-bearing C57BL/6 mice were also investigated by ELISA. The levels of IL-6, TNF-α, and IFN-γ in the CNS_D_ + L group were the highest and were 1.3, 1.2, and 1.2-fold higher than those in other groups (Fig. 5j–l).

### Gene expression analysis in vivo

**Fig. 6:**
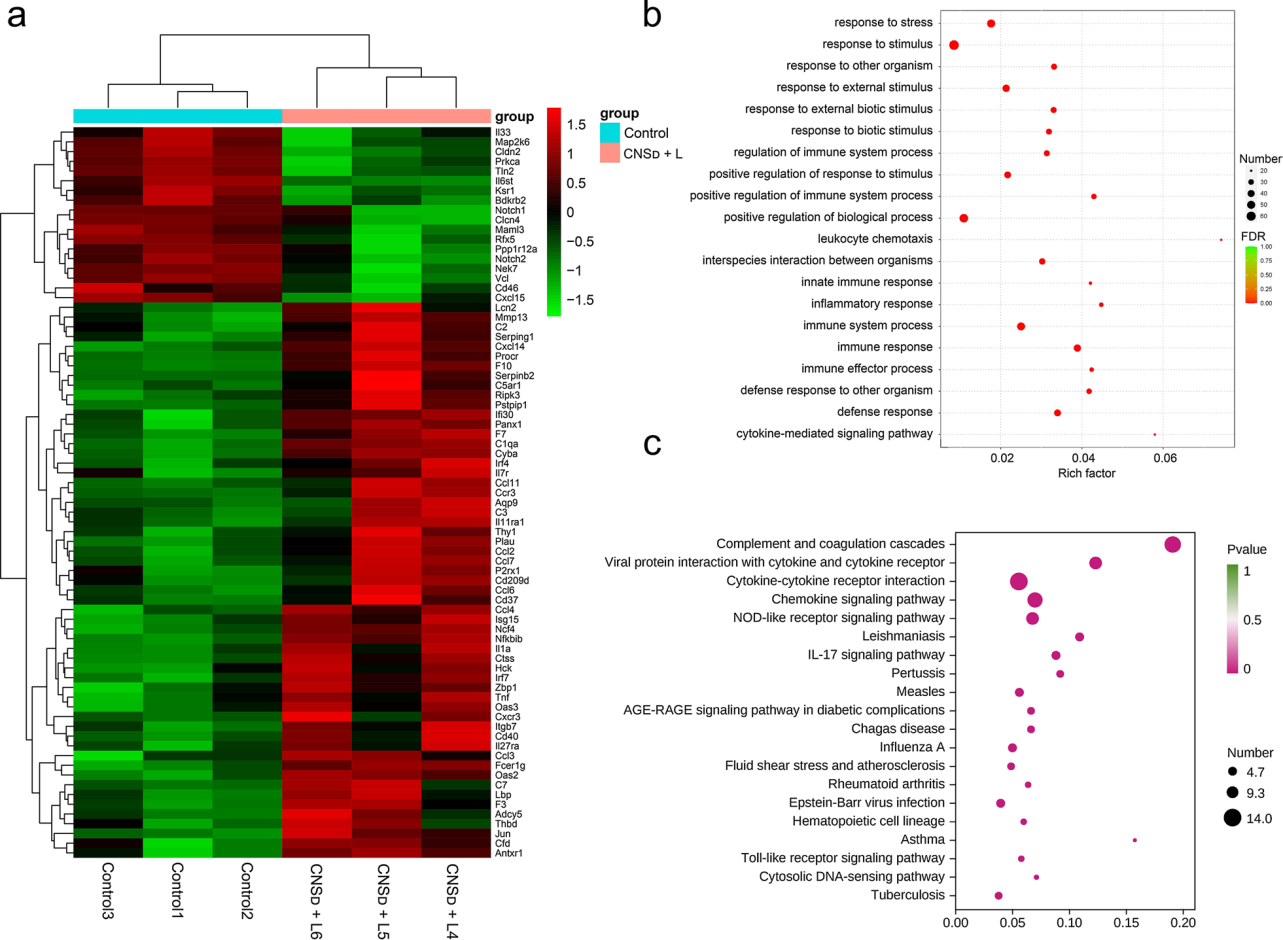
**a** Heat maps showing differential gene expression for screening immune function-related genes. **b**, **c** GO enrichment analysis (**b**) and KEGG enrichment analysis (**c**) of differentially upregulated genes associated with the immune process, with the 20 most significantly enriched categories listed

Transcriptomics analysis of CNS_D_ and PBS-treated mice was conducted to thoroughly investigate the mechanisms of immune activation at the genetic level in vivo. Analysis of immune-related genes across the whole genome revealed a total of 75 differentially expressed immune-related genes, among which, 57 were upregulated and 18 were downregulated (Additional file 1: Fig. S14). These differentially expressed immune-related genes were depicted in the heat map (Fig. 6a). Among the upregulated immune-related expressed genes, gene ontology (GO) process analyses and Kyoto Encyclopedia of Genes and Genomes (KEGG) pathway analyses were further performed to determine biological information, such as regulation pathway and biomolecular function. GO process analyses showed that the most enriched pathways included immune response, immune system response, cytokine-mediated signaling pathway, and regulation of immune response, which are closely related to immune activation (Fig. 6b). KEGG pathway analyses also indicated that several immune activation-related pathways, including Toll-like receptor signaling pathway and IL-17 signaling pathway, were enriched in CNS_D_-treated mice (Fig. 6c). In the CNS_D_-treated groups, several upregulated genes, including Tnf, Irf7, Jun, Ccl3, and Ccl4, were associated with the Toll-like receptor signaling pathway, which plays a major role in proinflammatory cytokine-induced DC maturation, implying that combined CNS_D_ and laser irradiation treatment may reawaken the antitumor immune response in the immunosuppressive tumor microenvironment [50, 51].

### Biocompatibility of CNS nanoparticles in vivo

The biocompatibility and biotoxicity of CNS nanoparticles were evaluated in vivo. No obvious behavioral abnormalities or weight loss were observed during the treatment of both 4T1 tumor-bearing Balb/c mice and Panc02 tumor-bearing mice (Additional file 1: Figs. S15, 16). Moreover, after treatment, H&E staining images showed that regions with necrosis or apoptosis were rarely detected in the murine major organs, including heart, liver, spleen, lung, and kidney in the different treatment groups (Additional file 1: Figs. S17, 18). Additionally, analysis of long-term biotoxicity in C57BL/6 mice revealed no significant differences in diversely vital blood parameters, and liver and kidney function indexes among the Control, D15, and D30 groups (Additional file 1: Fig. S19). The corresponding histological morphology in the H&E staining images remained normal at different treatment points (Control, D15, and D30). These results indicated the perfect biocompatibility of CNS nanoparticles for tumor immunotherapy in vivo.

## Conclusion

In conclusion, in the present study, a spatiotemporally controllable CNS nanoparticle was developed to achieve NIR-II photothermal-synergized immunotherapy via remotely photoactivated release of immune agents in specific areas. The CNS_D_ nanoparticles possessed a satisfactory photothermal conversion efficiency (≈ 32.6%) and enabled the effective accumulation in the murine tumor tissues after systemic administration. Under conditions of NIR-II photoirradiation, CNS_D_ rapidly elevated the localized temperature, leading to tumor ablation and induction of ICD, and precisely melted the protective lipid layer to release two antitumor immune agents in the tumor region. Levels of matured DCs and CD8^+^ T cells in CNS_D_-treated mice were increased by 2.4 and 2.3-fold, respectively, compared with those in the control group. Therefore, prepared CNS nanoparticles-mediated photothermal-synergized immunotherapy radically suppressed tumor growth in two types of murine models and also effectively inhibit pulmonary metastasis, suggesting a potentially novel strategy for safe and efficient photoactivated immunotherapy.

## Supplementary Information

**Additional file 1: Fig. S1.** Hydrodynamic diameters and PDI of CNS_D_ after storage in 1× PBS buffer (pH = 7.4) during 10 days (n = 3). **Fig. S2. **NIR images of CNS_0_, CNS_C_, CNS_J,_ and CNS_D_ ([CuS] = 100 µg mL^-1^) under the NIR-II laser irradiation (1064 nm, 1.0 W cm^−2^) for 5 min. **Fig. S3**. The time constant for heat transfer from the system is determined by applying the linear time data from the cooling period of (a) versus the negative natural logarithm of driving force temperature. **Fig. S4. **Release of CpG from CNS_D_ with or without laser irradiation (1064-nm, 1 W cm^−2^) for 5 min (the concentrations of CuS and CpG for CNS_D_ were 100 and 2.3 µg/mL, respectively). **Fig. S5.** Schematic illustration of the synthesis of ICG-loaded CNS nanoparticles (CNS_0_@ICG, CNS_C_@ICG, CNS_J_@ICG, CNS_D_@ICG) for tracing the nanoparticle trajectory. **Fig. S6**. UV-vis absorption spectrums of CNS_0_@ICG, CNS_C_@ICG, CNS_J_@ICG, and CNS_D_@ICG. **Fig. S7. **Fluorescence intensity of 4T1 cells treated with PBS (control) or various ICG-loaded CNS nanoparticles ([ICG] = 20 µg mL^−1^) for 24 h *via* flow cytometry. **Fig. S8. **Relative mean fluorescence intensity (MFI) of CRT in Panc02 cells after different treatments. **Fig. S9.** The NIR fluorescence imaging of xenograft Panc02tumor-bearing C57BL/6 living mice at 0, 8, 24, and 36 h after systemic administration of CNS@ICG through tail-vein administration (0.2 mL, [ICG] = 2 mg kg^-1^). The fluorescence images were collected with excitation at 710 nm and emission at 790 nm, and the tumors were marked by white circles. (b) The fluorescence intensity of tumor regions of mice at different post-injection times of (n = 3). **Fig. S10.** (a) NIR thermal photos of 4T1 tumor-bearing mice under laser irradiation at 24 h post-injection of PBS, CNS_0_, CNS_C_, CNS_J,_ and CNS_D_ through tail-vein injection (0.2 mL, the concentration of CuS = 300 µg/mL for CNS_0_, CNS_C_, CNS_J,_ and CNS_D_); (b) Temperature elevation curves of tumors in 4T1 tumor-bearing mice after administration of Control (PBS), CNS_0_, CNS_C_, CNS_J_ and CNS_D_ under NIR-II laser illumination (1064 nm, 1W cm^-2^, 5 min). **Fig. S11.** Gating strategies for flow cytometry assay of matured CD80^+^CD86^+^ DCs in tumor-draining lymph nodes of Panc02 tumor-bearing C57BL/6 mice. **Fig. S12.** Western-blot analysis of JQ1-induced downregulation of PD-L1 in Panc02 cells (200 nM of JQ1 and 100 ng/ml of IFN-γ). **Fig. S13. **Gating strategy for flow cytometry assay of CD4^+^ T cells and CD8^+^ T cells in distant tumors of Panc02 tumor-bearing C57BL/6 mice. **Fig. S14. **A volcano plot showing the up-regulated or insignificantly expressed or down-regulated genes when comparing the CNS_D_-treated group with the Control (PBS) group. **Fig. S15**. Body weights of Panc02 tumor-bearing C57BL/6 mice in different groups within 14 days of treatment (Control (PBS), CNS_0_, CNS_C_, CNS_J_ and CNS_D_, 0.2 mL, [CuS] = 300 μg/mL, n = 5). Fig**. S16**. Body weights of 4T1 tumor-bearing Balb/c mice in different groups within 14 days of treatment (Control (PBS), CNS_0_, CNS_C_, CNS_J_ and CNS_D_, 0.2 mL, [CuS] = 300 μg/mL, n = 5). **Fig. S17.** Representative histological H&E staining images of major organs (heart, liver, spleen, lung, and kidney) were collected from Panc02 tumor-bearing C57BL/6 mice in different treatment groups at the end of treatment (Control (PBS), CNS_0_, CNS_C_, CNS_J_ and CNS_D_, 0.2 mL, [CuS] = 300 μg/mL). The scale bar represents 50 μm. **Fig. S18.** Representative histological H&E staining photos of major organs (heart, liver, spleen, lung, and kidney) in healthy C57BL/6 mice before treatment (Control) and after tail-intravenous injection of CNS_D_ (0.2 mL, [CuS] = 00 μg/mL) for 15 (Day 15) and 30 days (Day 30). The scale bar represents 50 μm. **Fig. S19. **The levels of (a) alanine aminotransferase (ALT), (b) aspartate aminotransferase (AST), (c) γ-glutamyl transpeptidase (GGT), (d) urea, (e) creatinine (CREA), (f) red blood cells (RBC), (g) hemoglobin (HGB), (h) mean corpuscular hemoglobin (MCH), (i) hemoglobin concentration (MCHC), (j) red cell distribution width (RDW-SD), (k) red cell volume distribution width (RDW-CV), (l) platelet (PLT), (m) plateletcrit (PCT), (n) mean platelet volume (MPV), and (o) platelet distribution width (PDW) in the blood of healthy C57BL/6 mice before treatment (D0) and after tail-intravenous administration of CNS_D_ (0.2 mL, [CuS] = 300 μg/mL) for 15 (D15), and 30 days (D30) (n = 3).

## Data Availability

All data analyzed in this study are available in the main text and supporting information.

## Abbreviations

NIR-II: Second near-infrared
CuS: Copper sulfide
TLR-9: Toll-like receptor-9
PD-L1: Programmed death-ligand 1
ICD: Immunogenic cell death
ICBs: Immune checkpoint inhibitors
PD-1: Programmed death 1
PD-L1: Programmed death ligand 1
CTLA-4: Cytotoxic T lymphocyte-associated protein 4
FDA: Food and Drug Administration
PTT: Photothermal therapy
DAMPs: Damage-associated molecular patterns
DCs: Dendritic cells
MPE: Maximum permissible exposure
TLR-9: Toll-like receptor-9
DPPC: 1,2-Dipalmitoyl-*sn-*glycerol-3-phosphocholine
DSPE–PEG_2k_: 1,2-Distearoyl-*sn-*glycero-3-phosphoethanolamine-poly(ethylene glycol)2000
APCs: Antigen-presenting cells
ANOVA: One-way analysis of variance
EE: Encapsulation efficiency
LC: Loading capacity
TEM: Transmission electron microscopy
DLS: Dynamic light scattering
PBS: Phosphate-buffered saline
UV–vis: Ultraviolet–visible
ICG: Indocyanine green
CRT: Calreticulin
MFI: Mean fluorescence intensity
BMDCs: Bone marrow-derived dendritic cells
IL-6: Interleukin-6
TNF-α: Tumor necrosis factor-α
H&E: Hematoxylin and eosin
TUNEL: Terminal deoxynucleotidyl transferase dUTP nick end labeling
ATP: Adenosine triphosphate
HMGB1: High mobility group box 1 protein
IFN-γ: Interferon-gamma
GO: Gene ontology
KEGG: Kyoto Encyclopedia of Genes and Genomes

## Declarations

### Comment for publication

The authors agreed to be published.
