# Supplementary material for: Second near-infrared photothermal-amplified immunotherapy using photoactivatable composite nanostimulators
Source: J Nanobiotechnology. 2021 Dec 20;19:433. doi: 10.1186/s12951-021-01197-5 (PMC8686222; doi:10.1186/s12951-021-01197-5)
Supplement: Supplementary file 1 — Additional file 1: Fig. S1. Hydrodynamic diameters and PDI of CNSD after storage in 1× PBS buffer (pH = 7.4) during 10 days (n = 3). Fig. S2. NIR images of CNS0, CNSC, CNSJ, and CNSD ([CuS] = 100 µg mL-1) under the NIR-II laser irradiation (1064 nm, 1.0 W cm−2) for 5 min. Fig. S3. The time constant for heat transfer from the system is determined by applying the linear time data from the cooling period of (a) versus the negative natural logarithm of driving force temperature. Fig. S4. Release of CpG from CNSD with or without laser irradiation (1064-nm, 1 W cm−2) for 5 min (the concentrations of CuS and CpG for CNSD were 100 and 2.3 µg/mL, respectively). Fig. S5. Schematic illustration of the synthesis of ICG-loaded CNS nanoparticles (CNS0@ICG, CNSC@ICG, CNSJ@ICG, CNSD@ICG) for tracing the nanoparticle trajectory. Fig. S6. UV-vis absorption spectrums of CNS0@ICG, CNSC@ICG, CNSJ@ICG, and CNSD@ICG. Fig. S7. Fluorescence intensity of 4T1 cells treated with PBS (control) or various ICG-loaded CNS nanoparticles ([ICG] = 20 µg mL−1) for 24 h via flow cytometry. Fig. S8. Relative mean fluorescence intensity (MFI) of CRT in Panc02 cells after different treatments. Fig. S9. The NIR fluorescence imaging of xenograft Panc02tumor-bearing C57BL/6 living mice at 0, 8, 24, and 36 h after systemic administration of CNS@ICG through tail-vein administration (0.2 mL, [ICG] = 2 mg kg-1). The fluorescence images were collected with excitation at 710 nm and emission at 790 nm, and the tumors were marked by white circles. (b) The fluorescence intensity of tumor regions of mice at different post-injection times of (n = 3). Fig. S10. (a) NIR thermal photos of 4T1 tumor-bearing mice under laser irradiation at 24 h post-injection of PBS, CNS0, CNSC, CNSJ, and CNSD through tail-vein injection (0.2 mL, the concentration of CuS = 300 µg/mL for CNS0, CNSC, CNSJ, and CNSD); (b) Temperature elevation curves of tumors in 4T1 tumor-bearing mice after administration of Control (PBS), CNS0, CNSC, CNSJ an [file 12951_2021_1197_MOESM1_ESM.docx]

Additional file 1

Second near-infrared photothermal-amplified immunotherapy using photoactivatable composite nanostimulators

Haitao Sun^1†^, Tianzhu Yu^1†^, Xin Li^3†^, Yanyang Lei^1^, Jianke Li^1^, Xiuhui Wang^4*^, Peike Peng^5*^, Dalong Ni^6^, Xiaolin Wang^1*^, Yu Luo^2*^

^1^Department of Interventional Radiology, Zhongshan Hospital, Fudan University, Shanghai Institute of Medical Imaging, No. 180 Fenglin Road, Xuhui District, Shanghai 200032, China

^2^Frontier Institute of Medical & Pharmaceutical Science and Technology, School of Chemistry and Chemical Engineering, Shanghai University of Engineering Science, No. 333 Longteng Road, Shanghai 201620, P. R. China

^3^Institute for Technical and Macromolecular Chemistry, RWTH Aachen University, Worringerweg 2, 52074 Aachen, Germany

^4^Institute of Translational Medicine, Shanghai University, Shanghai 200011, P. R. China

^5^ School of Basic Medical Sciences, Shanghai University of Traditional Chinese Medicine, Shanghai, P. R. China

^6^ Department of Orthopaedics, Shanghai Key Laboratory for Prevention and Treatment of Bone and Joint Diseases, Shanghai Institute of Traumatology and Orthopaedics, Ruijin Hospital, Shanghai Jiao Tong University School of Medicine, 197 Ruijin 2nd Road, Shanghai 200025, P. R. China.

^*^Correspondence: fduwangxiaolin@hotmail.com (Xiaolin Wang), blackrabbit@shu.edu.cn (Xiuhui Wang), pengpeike@shutcm.edu.cn (Peike Peng), and yuluo@sues.edu.cn (Yu Luo)

^†^Haitao Sun, Tianzhu Yu and Xin Li contributed equally to this work.

# Material and methods

**Materials.** Bovine serum albumin (BSA), copper (II) chloride dihydrate (CuCl_2_·2H_2_O), sodium sulfide nonahydrate (Na_2_S·9H_2_O), and sodium hydroxide (NaOH) were obtained from Sigma–Aldrich Co. (Shanghai, China). DPPC and DSPE–PEG_2k_ were purchased from Ananti Polar Lipids, Inc. (Alabaster, AL, USA). ICG was obtained from Shanghai Aicheng Biological Technology Co., Ltd (Shanghai, China). JQ1 was purchased from MedChemExpress (Monmouth Junction, NJ, USA). CpG 1826 (5′-TCC ATG ACG TTC CTG ACG TT-3′) and Cy5.5-CpG 1826 (5′-TCC ATG ACG TTC CTG ACG TT-3′-Cy5.5) were synthesized at Sangon Biotechnology Co., Ltd (Shanghai, China). Antibodies, including CD45-BV605 (Biolegend, Clone: 30-F11, Catalog No. 103140), CD3-APC/Cyanine7 (Biolegend, Clone: 17A2, Catalog No. 100222), CD4-FITC (Biolegend, Clone: GK1.5, Catalog No. 100406), CD8-PE (Biolegend, Clone: 53-5.8, Catalog No. 140408), CD11c-FITC (Biolegend, Clone: N418, Catalog No. 117306), CD80-PE (Biolegend, Clone: 16-10A1, Catalog No. 104708), and CD86-APC (Biolegend, Clone: GL-1, Catalog No. 105012) were purchased from Biolegend, Inc. Anti-calreticulin antibody (Catalog No. ab227444), recombinant anti-HMGB1 antibody (Catalog No. ab79823), and recombinant anti-PD-L1 antibody (Catalog No. ab213480) were all purchased from Abcam. Fetal bovine serum (FBS), Dulbecco’s Modified Eagle’s Medium (DMEM), RPMI 1640, penicillin, streptomycin, and 0.25% trypsin-EDTA were purchased from Gibco (New York, USA). 2-Mercaptoethanol and granulocyte-macrophage colony-stimulating factor were purchased from Shanghai Aladdin Biochemical Technology Co., Ltd. Cell counting kit-8 (CCK-8) were purchased from 7Sea Pharmatech, Co., Ltd (Shanghai, China). Mouse IFN-γ, TNF-α, and IL-6 ELISA kits were purchased from Shanghai Suran Biotechnology Co., Ltd (Shanghai, China).

**Characterization.** Transmission electron microscope (TEM) images were obtained using a JEM 2100F transmission electron microscope (JEOL, Japan). Zetasizer Nano series (Nano ZS90, Malvern) was used to analyze hydrodynamic diameters and zeta potentials. A 3600 spectrometer (Shimadzu, Japan) was used to record the absorption spectra of various nanoparticles. High-performance liquid chromatography (HPLC) was using the LC-20A Prominence UFLC system (Shimadzu, Japan). A FLIR 225s IR thermal camera was used to obtain the thermal images. An IVIS imaging system (IVIS-CT machine, PerkinElmer, MA, USA) was used to obtain fluorescence images *in vivo*. A CytoFLEX LX flow cytometer (Beckman Coulter, Inc) was used for flow cytometric analysis.

**Evaluation of CNS photothermal properties.** Aqueous solutions of various CNS (0.2 mL) at CuS concentrations of 100 μg/mL were exposed to a NIR laser (1 W cm^−2^) to evaluate the photothermal performance of CNS nanoparticles. First, CNS aqueous solutions were irradiated with a NIR-II laser to increase to the maximum temperature and the laser was then artificially turned off so the temperature of the solution naturally dropped to near room temperature. All processes were recorded using a FLIR 225s IR thermal camera. Next, the photothermal stability of CNS nanoparticles was assessed. Five cycles of heating and cooling for CNS solutions were recorded using a FLIR 225s IR thermal camera. The corresponding photothermal conversion efficiency of four CNS nanoparticles was calculated as previously described.[1]

**Photothermally activated release of JQ1 and CpG from CNS.** CNS_D_ in 1 × PBS buffer was irradiated for 5 min (1 W cm^−2^) and placed into a dialysis bag (MW = 3,500) to determine the photothermally activated release of JQ1. The dialysis bag was stored in a 50-mL centrifuge tube containing 20 mL of 1 × PBS (pH = 7.4, 0.5% Tween-20) and placed in a constant-temperature shaker (37°C, 120 rpm). Next, 2 mL of external liquid was removed at different time points (0, 5, 10, 20, 40, and 60 min) and an equal volume of new PBS solution (pH = 7.4, 0.5% Tween-20) was added. CNS_D_ without photoirradiation was considered the control group. The JQ1 content in the collected PBS solutions was then measured using HPLC. Then, the photothermally activated release of CpG (Cy5.5-CpG) from CNS_D_ was further investigated. The samples were processed and collected as described above. The UV-vis spectrophotometry was used to measure the CpG content. All assays were conducted three times.

**Cell culture.** Panc02 murine pancreatic cancer cells and 4T1 murine breast tumor cells were used in the present study. Panc02 cells and 4T1 cells were cultured in complete DMEM and RMPI-1640, respectively, containing 10% FBS, 100 U mL^−1^ penicillin, and 0.1 mg mL^−1^ streptomycin. Both cell lines were cultured at 37 ℃ under humidified conditions with a 5% CO_2_ supply.

***In vitro* DC maturation.** BMDCs were extracted from eight-week-old C57BL/6 mice as described previously.[2, 3] Following different treatments, Panc02 cancer cells were implanted in the upper compartment of a transwell system and then co-cultured with BMDCs implanted in the lower compartment for 24 h. The obtained DCs were then stained with anti-CD11c FITC, anti-CD80 PE, and anti-CD86 APC and examined using FCM (Beckman Coulter). After treatment, the obtained DC suspensions were collected and examined using ELISA kits to examine the content of proinflammatory cytokines, including IL-6 and TNF-α.

**PD-L1 inhibition by JQ1 *in vitro*.** To investigate the effect of JQ1 on PD-L1 expression *in vitro*, Panc02 cells were incubated in 6 well plates at a density of 2 × 10^5^ cells per well for 24 h. To simulate the expression of PD-L1, the cells were then treated with IFN-γ (100 ng/mL) for 24 h. Next, the cells were respectively treated with free JQ1 (200 nM) and released JQ1 (CNS_J_) (the CNS_J_ was pretreated with laser irradiation, [JQ1] = 200 nM) for another 24 h. Finally, the cells were collected to analyze the expression of PD-L1 by western blot.

**CNS-induced ICD *in vitro*.** CRT expression, which is a representative biomarker of ICD, was measured using flow cytometric assay to determine CNS-induced ICD in vitro as follows. Panc02 cells were cultured in 12-well plates at a density of 2 × 10^6^/well for 24 h. The cells were then treated with PBS or 50 μg/mL CNS_0_, CNS_C_, CNS_J_, and CNS_D_ for 24 h. The cells were then washed three times with PBS and a new medium was added. Next, cells treated with four types of CNS nanoparticles were respectively exposed under the NIR-II laser (1064 nm, 1.0 W cm^−2^, 5 min) and subsequently cultured for 24 h. PBS and CNS_D_-treated cells, which served as the no laser-treatment groups, were simultaneously cultured for 24 h without photoirradiation. The cells were collected after different treatments and stained with an anti-calreticulin antibody (ab227444) and analyzed using flow cytometry (Beckman Coulter).

**Evaluation of intratumoral ICD *in vivo***. Intratumoral ATP levels were investigated. Panc02 tumor-bearing C57BL/6 mice were randomly divided into six groups: control (PBS); CNS_0_ + L; CNS_C_ + L; CNS_J_ + L; CNS_D_; and CNS_D_ + L (n = 3). The mice in each group were intravenously administered with 0.2 mL of PBS or 300 μg/mL CNS_0_, CNS_C_, CNS_J_, and CNS_D_. Each primary tumor was then photoirradiation using a 1064-nm laser (1 W cm^−2^) for 5 min at 24 h post-injection. On day 2 post-treatment, the mice were sacrificed and the tumors were collected. The tumors were ground to determine the ATP levels using an ATP kit according to the manufacturer’s protocol. At day 2 post-treatment, the primary tumors in each group were collected and stained with anti-calreticulin antibody (Catalog No. ab227444) and recombinant anti-HMGB1 antibody (Catalog No. ab79823) for immunohistochemical and immunofluorescent analysis, respectively, to assess the intratumoral levels of CRT and HMGB1.

***In vivo* evaluation of DC maturation****.** Panc02 tumor-bearing C57BL/6 mice were randomly divided into six groups: control (PBS); CNS_0_ + L; CNS_C_ + L; CNS_J_ + L; CNS_D_; CNS_D_ + L (n = 3). The mice in each group were intravenously administered with 0.2 mL of PBS or 300 μg/mL CNS_0_, CNS_C_, CNS_J_, and CNS_D_. Each primary tumor was photoirradiation using a 1064-nm laser (1 W cm^−2^) for 5 min at 24 h post-injection. At day 3 post-treatment, the inguinal tumor-draining lymph nodes were collected from each mouse and homogenized into single-cell suspension (1 × PBS), followed by staining for CD45-BV605 (Biolegend, Clone: 30-F11, Catalog No. 103140), CD11c-FITC (Biolegend, Clone: N418, Catalog No. 117306), CD80-PE (Biolegend, Clone: 16-10A1, Catalog No. 104708), and CD86-APC (Biolegend, Clone: GL-1, Catalog No. 105012) according to the manufacture’s protocol. The stained cells were then examined using A CytoFLEX LX flow cytometer (Beckman Coulter, Inc).

**Evaluation of serum cytokine levels *in vivo*.** Panc02 tumor-bearing C57BL/6 mice were randomly divided into six groups: control (PBS); CNS_0_ + L; CNS_C_ + L; CNS_J_ + L; CNS_D_; and CNS_D_ + L (n = 3). The mice in each group were intravenously administered with 0.2 mL of PBS or 300 μg/mL CNS_0_, CNS_C_, CNS_J_, and CNS_D_. Each primary tumor was photoirradiation using a 1064 nm laser (1 W cm^−2^) for 5 min at 24 h post-injection. On day 3 post-treatment, serum was collected from each mouse. The levels of TNF-α, IFN-γ, and IL-6 in the serum samples were determined using ELISA kits according to the manufacturer's protocols.

***In vivo* assessment of intratumoral infiltration of T lymphocytes.** Panc02 tumor-bearing C57BL/6 mice were randomly divided into six groups: Control (PBS); CNS_0_ + L; CNS_C_ + L; CNS_J_ + L; CNS_D_; and CNS_D_ + L (n = 3) to examine the intratumoral infiltration of T lymphocytes. The mice in each group were intravenously administered with 0.2 mL of PBS or 300 μg/mL CNS_0_, CNS_C_, CNS_J_, and CNS_D._ Each primary tumor was photoirradiation using a 1064-nm laser (1 W cm^−2^) for 5 min at 24 h post-injection. At 10 days post-treatment, distant tumors (n = 3) were collected and homogenized into single-cell suspension (1 × PBS). The cells were stained for CD45-BV605 (Biolegend, Clone: 30-F11, Catalog No. 103140), CD3-APC/Cyanine7 (Biolegend, Clone: 17A2, Catalog No. 100222), CD4-FITC (Biolegend, Clone: GK1.5, Catalog No. 100406), and CD8-PE (Biolegend, Clone: 53-5.8, Catalog No. 140408) and examined using an A CytoFLEX LX flow cytometer (Beckman Coulter, Inc).

**References**

1. Zhou J, Jiang Y, Hou S, Upputuri PK, Wu D, Li J, et al. Compact Plasmonic Blackbody for Cancer Theranosis in the Near-Infrared II Window. ACS Nano. 2018;12:2643-2651.

2. Xu L, Liu Y, Chen Z, Li W, Liu Y, Wang L, et al. Morphologically Virus-Like Fullerenol Nanoparticles Act as the Dual-Functional Nanoadjuvant for HIV-1 Vaccine. Adv Mater. 2013;25:5928-5936.

3. Lutz MB, Kukutsch N, Ogilvie AL, Rossner S, Koch F, Romani N, et al. An advanced culture method for generating large quantities of highly pure dendritic cells from mouse bone marrow. J Immunol Methods. 1999;223:77-92.


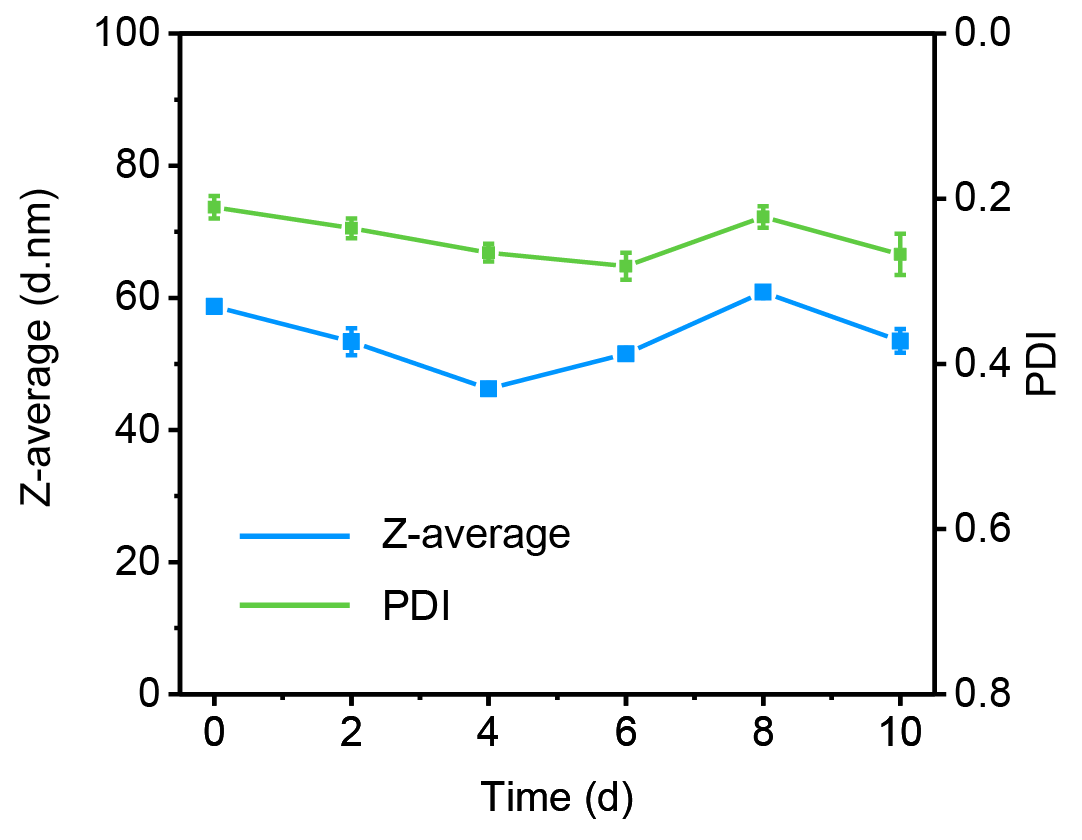


**Figure S1.** Hydrodynamic diameters and PDI of CNS_D_ after storage in 1× PBS buffer (pH = 7.4) during 10 days (n = 3).


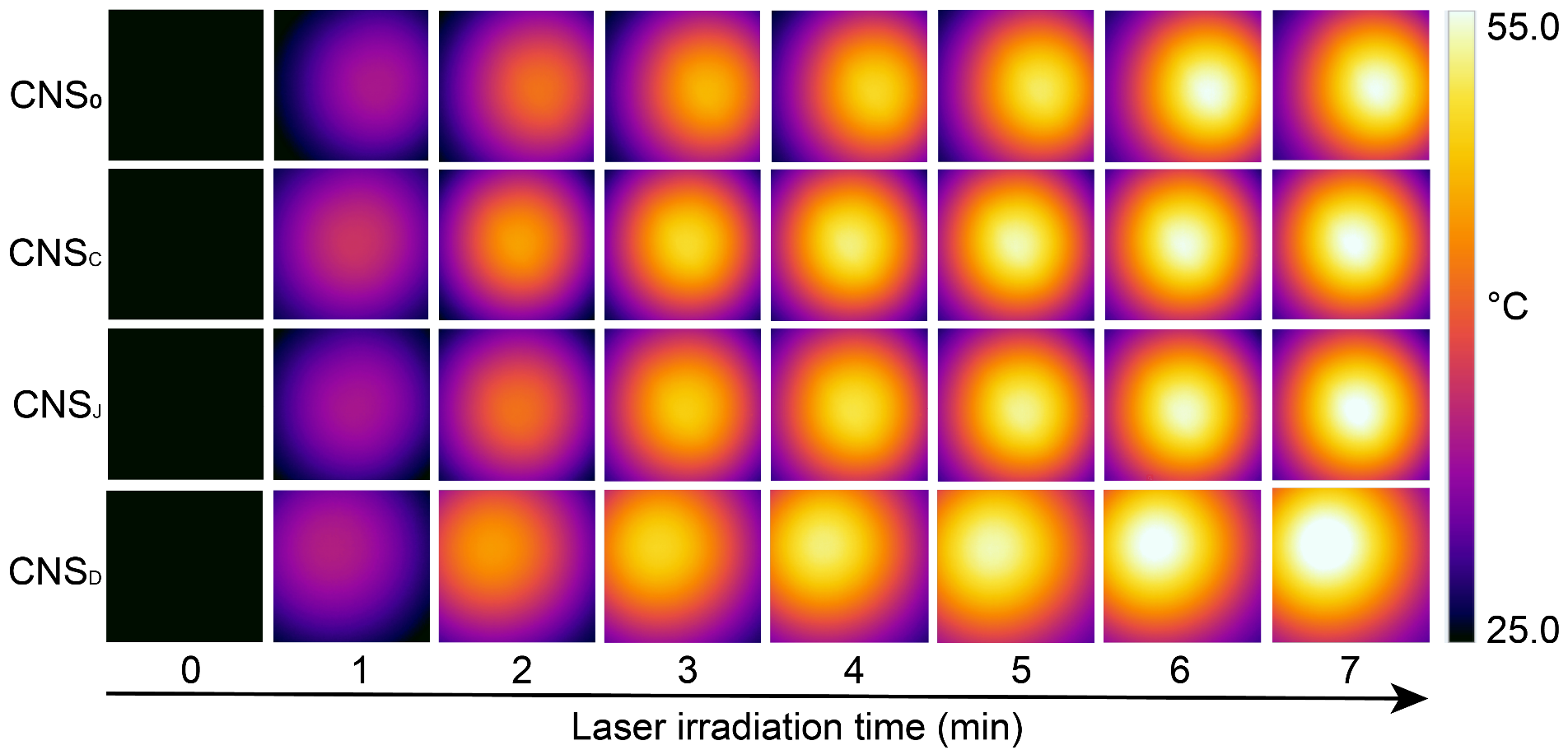


**Figure S2.** NIR images of CNS_0_, CNS_C_, CNS_J,_ and CNS_D_ ([CuS] = 100 µg mL^-1^) under the NIR-II laser irradiation (1064 nm, 1.0 W cm^−2^) for 5 min.


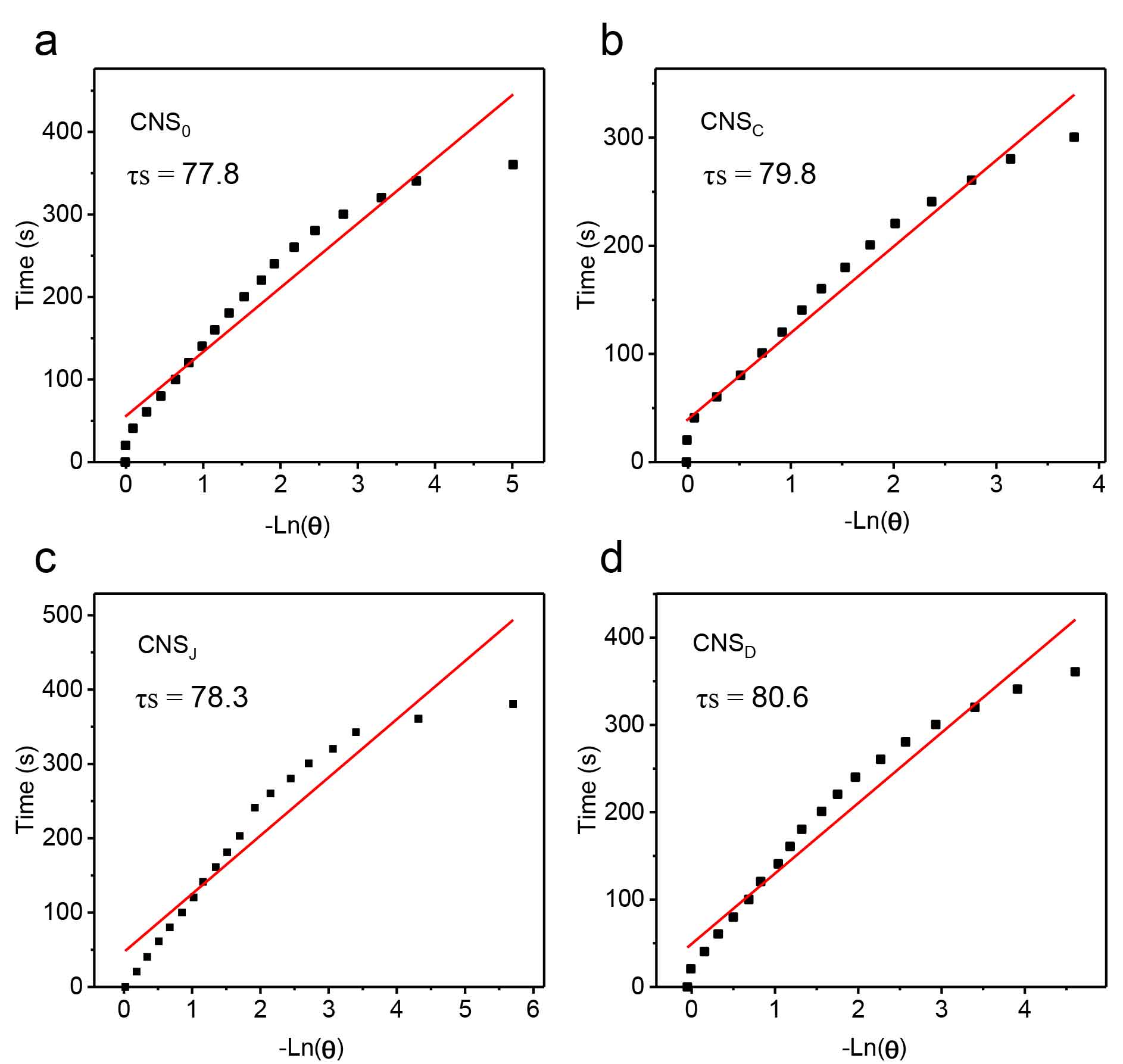


**Figure S3.** The time constant for heat transfer from the system is determined by applying the linear time data from the cooling period of (a) versus the negative natural logarithm of driving force temperature.


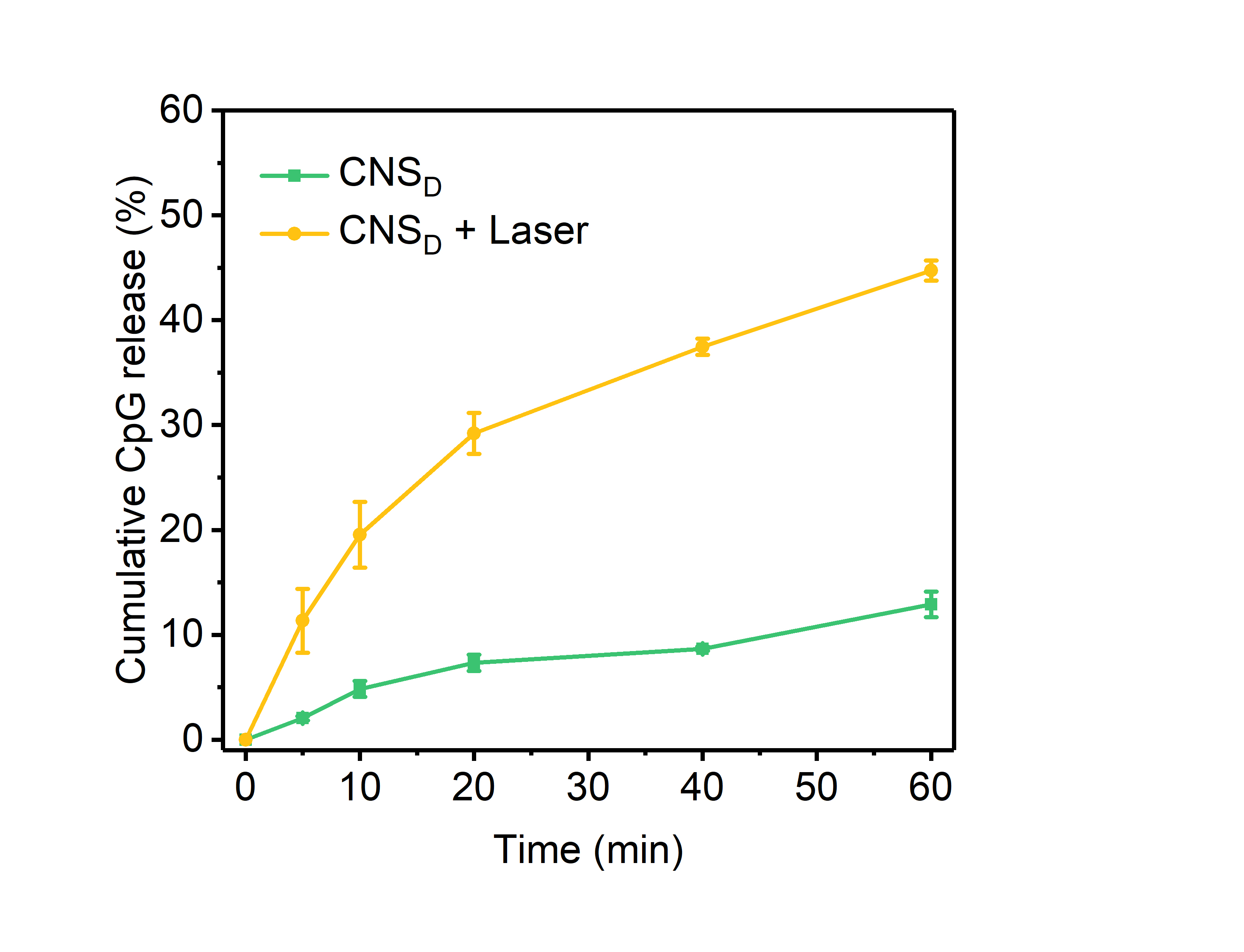


**Figure S4.** Release of CpG from CNS_D_ with or without laser irradiation (1064-nm, 1 W cm^−2^) for 5 min (the concentrations of CuS and CpG for CNS_D_ were 100 and 2.3 µg/mL, respectively).


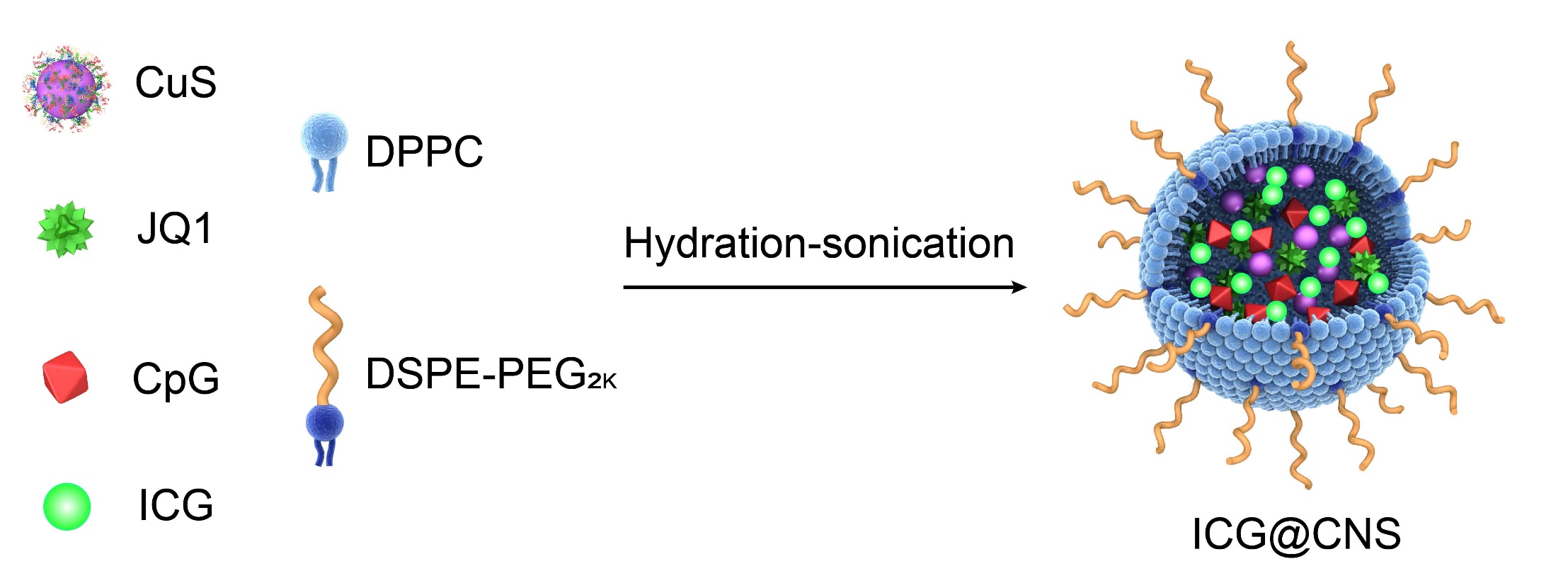


**Figure S5.** Schematic illustration of the synthesis of ICG-loaded CNS nanoparticles (CNS_0_@ICG, CNS_C_@ICG, CNS_J_@ICG, CNS_D_@ICG) for tracing the nanoparticle trajectory.


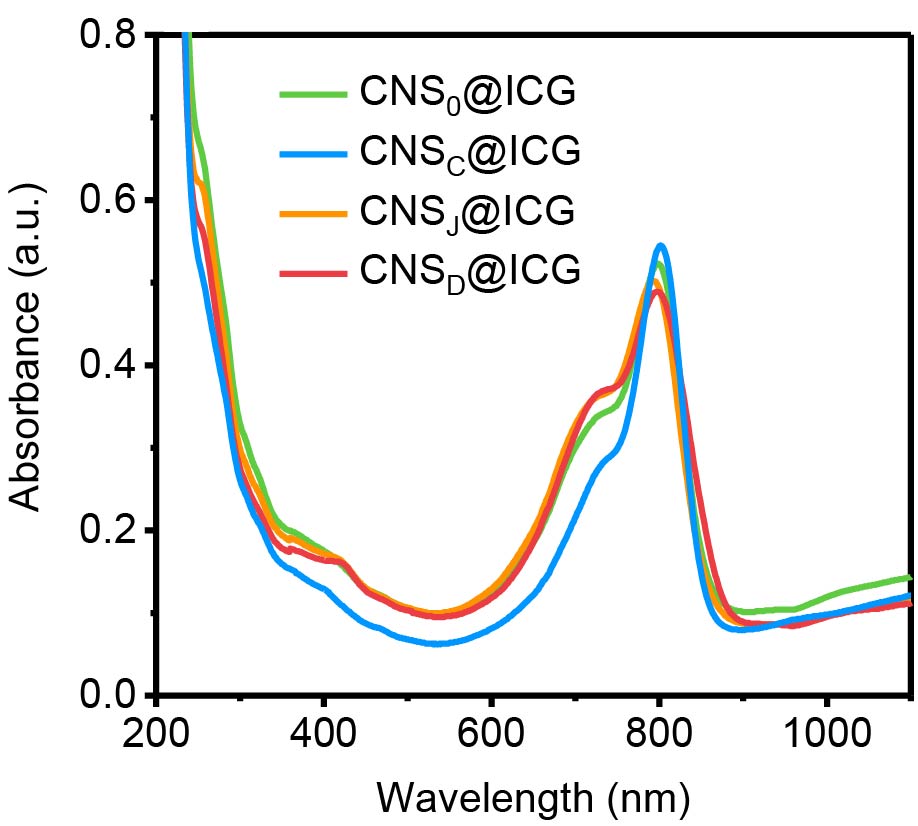


**Figure S6.** UV-vis absorption spectrums of CNS_0_@ICG, CNS_C_@ICG, CNS_J_@ICG, and CNS_D_@ICG.


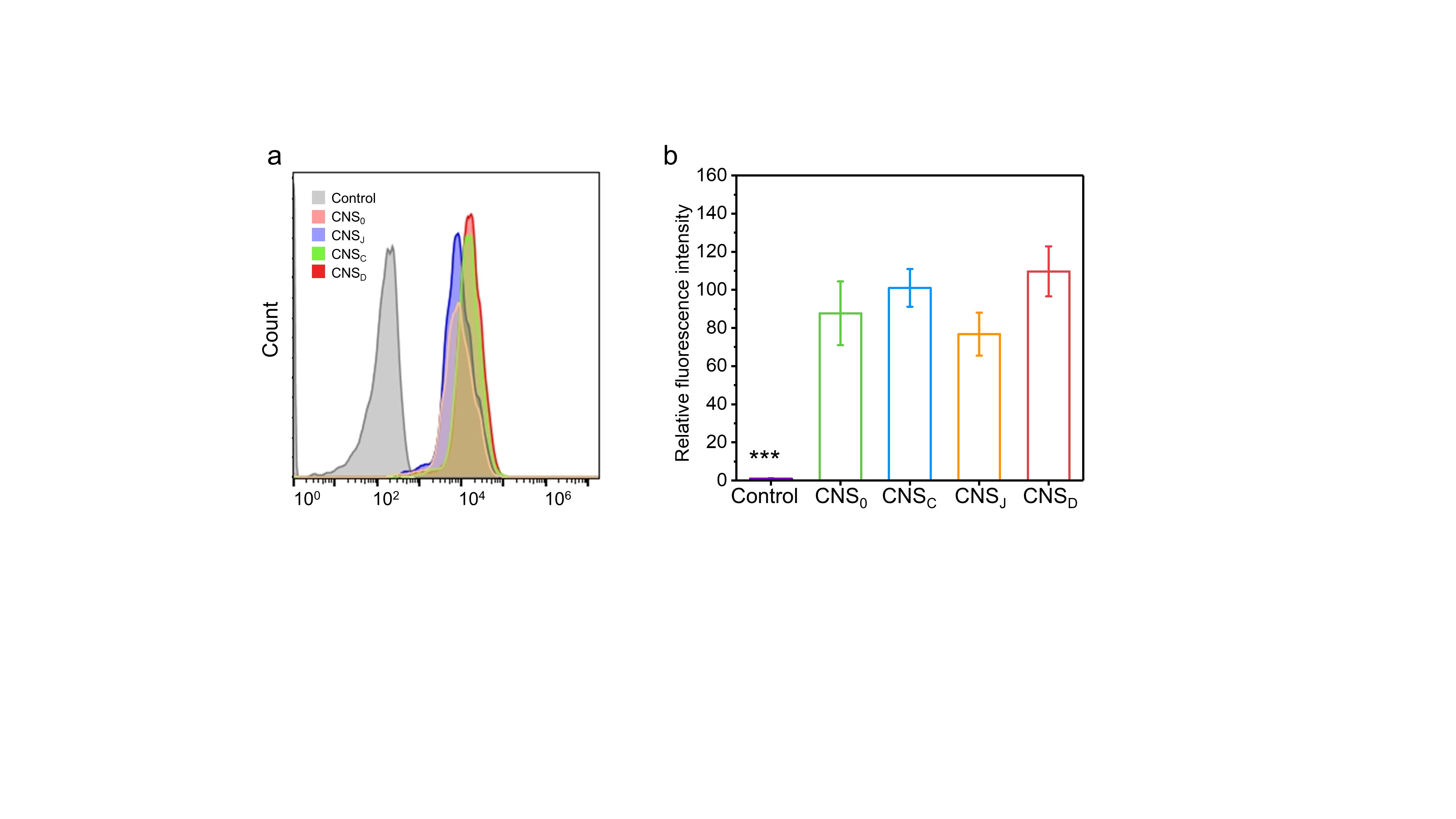


**Figure S7.** Fluorescence intensity of 4T1 cells treated with PBS (control) or various ICG-loaded CNS nanoparticles ([ICG] = 20 µg mL^−1^) for 24 h *via* flow cytometry.


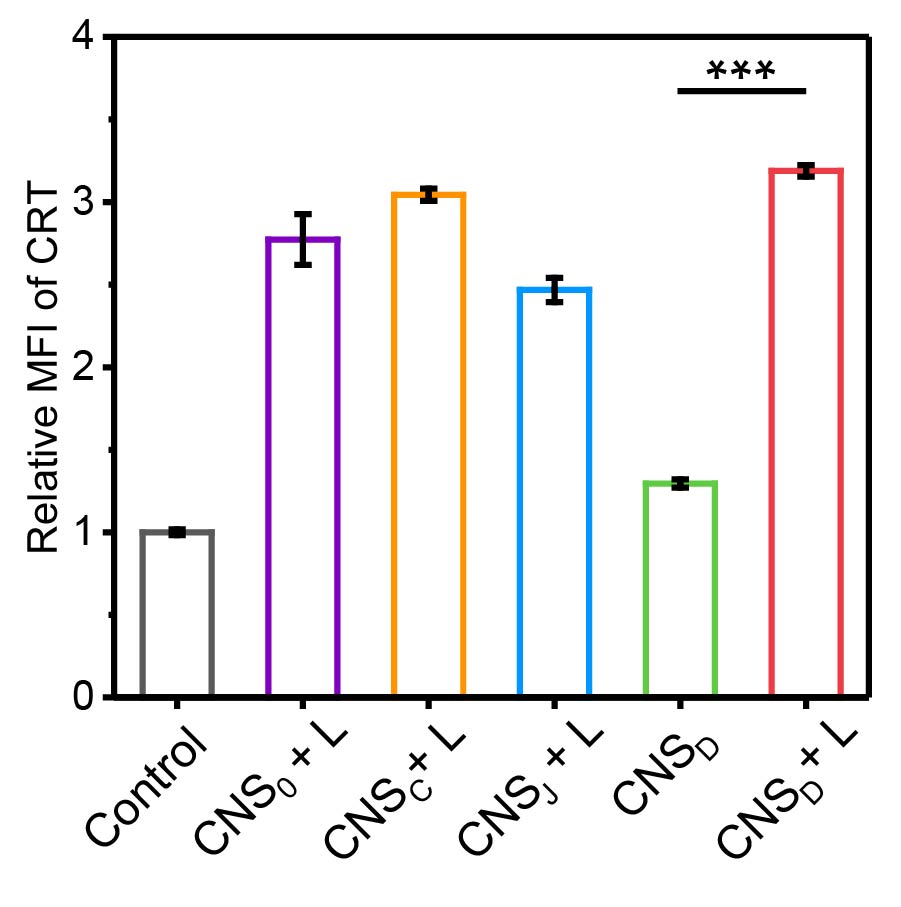


**Figure S8.** Relative mean fluorescence intensity (MFI) of CRT in Panc02 cells after different treatments.


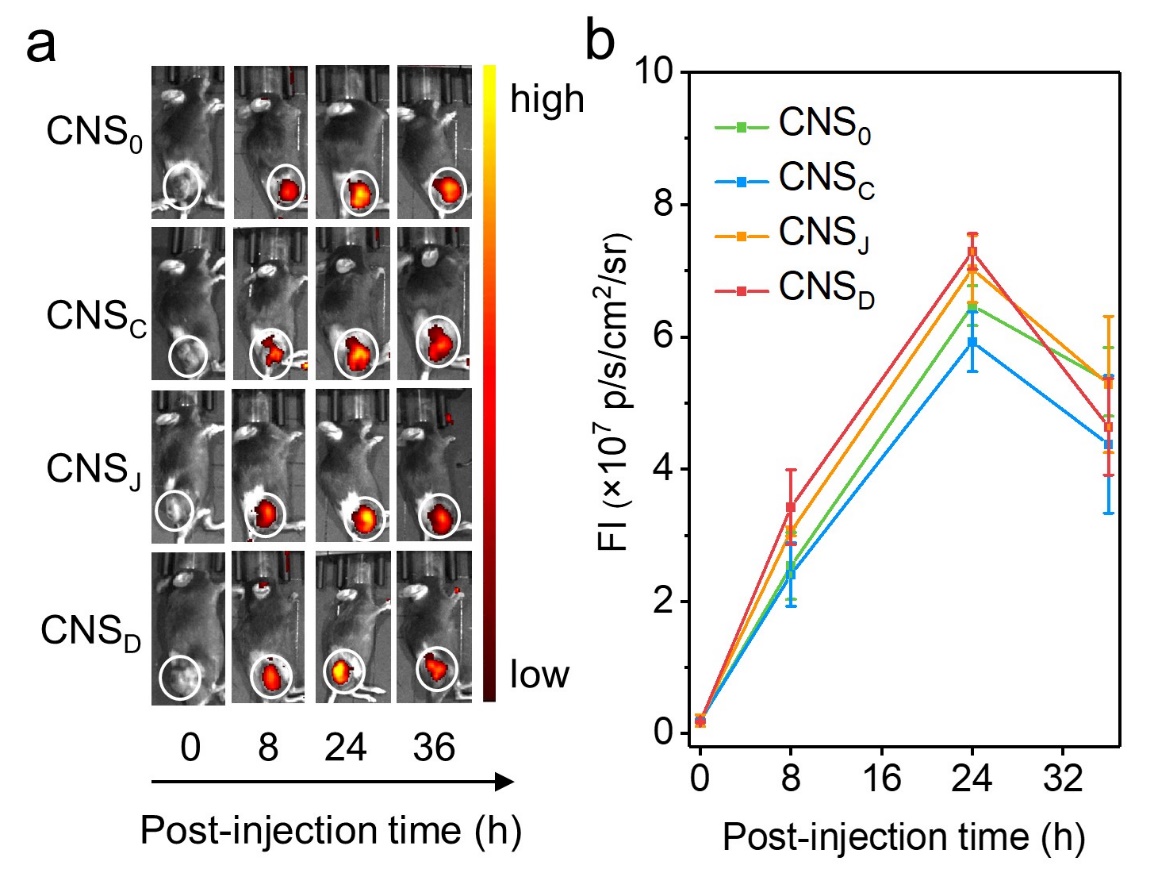


**Figure S9.** The NIR fluorescence imaging of xenograft Panc02tumor-bearing C57BL/6 living mice at 0, 8, 24, and 36 h after systemic administration of CNS@ICG through tail-vein administration (0.2 mL, [ICG] = 2 mg kg^-1^). The fluorescence images were collected with excitation at 710 nm and emission at 790 nm, and the tumors were marked by white circles. (b) The fluorescence intensity of tumor regions of mice at different post-injection times of (n = 3).


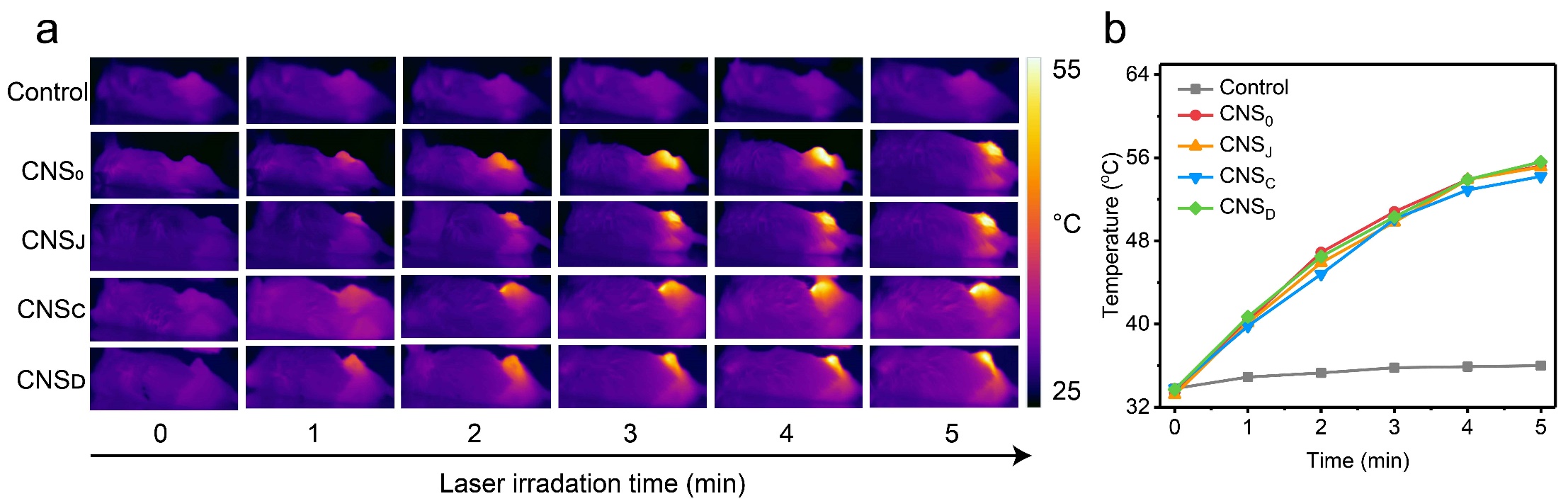


**Figure S10.** (a) NIR thermal photos of 4T1 tumor-bearing mice under laser irradiation at 24 h post-injection of PBS, CNS_0_, CNS_C_, CNS_J,_ and CNS_D_ through tail-vein injection (0.2 mL, the concentration of CuS = 300 µg/mL for CNS_0_, CNS_C_, CNS_J,_ and CNS_D_); (b) Temperature elevation curves of tumors in 4T1 tumor-bearing mice after administration of Control (PBS), CNS_0_, CNS_C_, CNS_J_ and CNS_D_ under NIR-II laser illumination (1064 nm, 1W cm^-2^, 5 min).


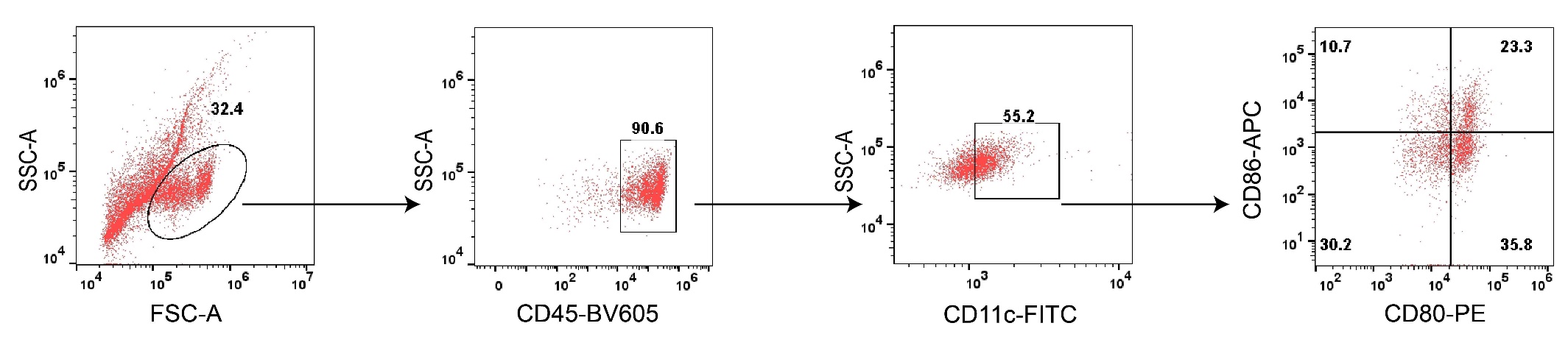


**Figure S11.** Gating strategies for flow cytometry assay of matured CD80^+^CD86^+^ DCs in tumor-draining lymph nodes of Panc02 tumor-bearing C57BL/6 mice.


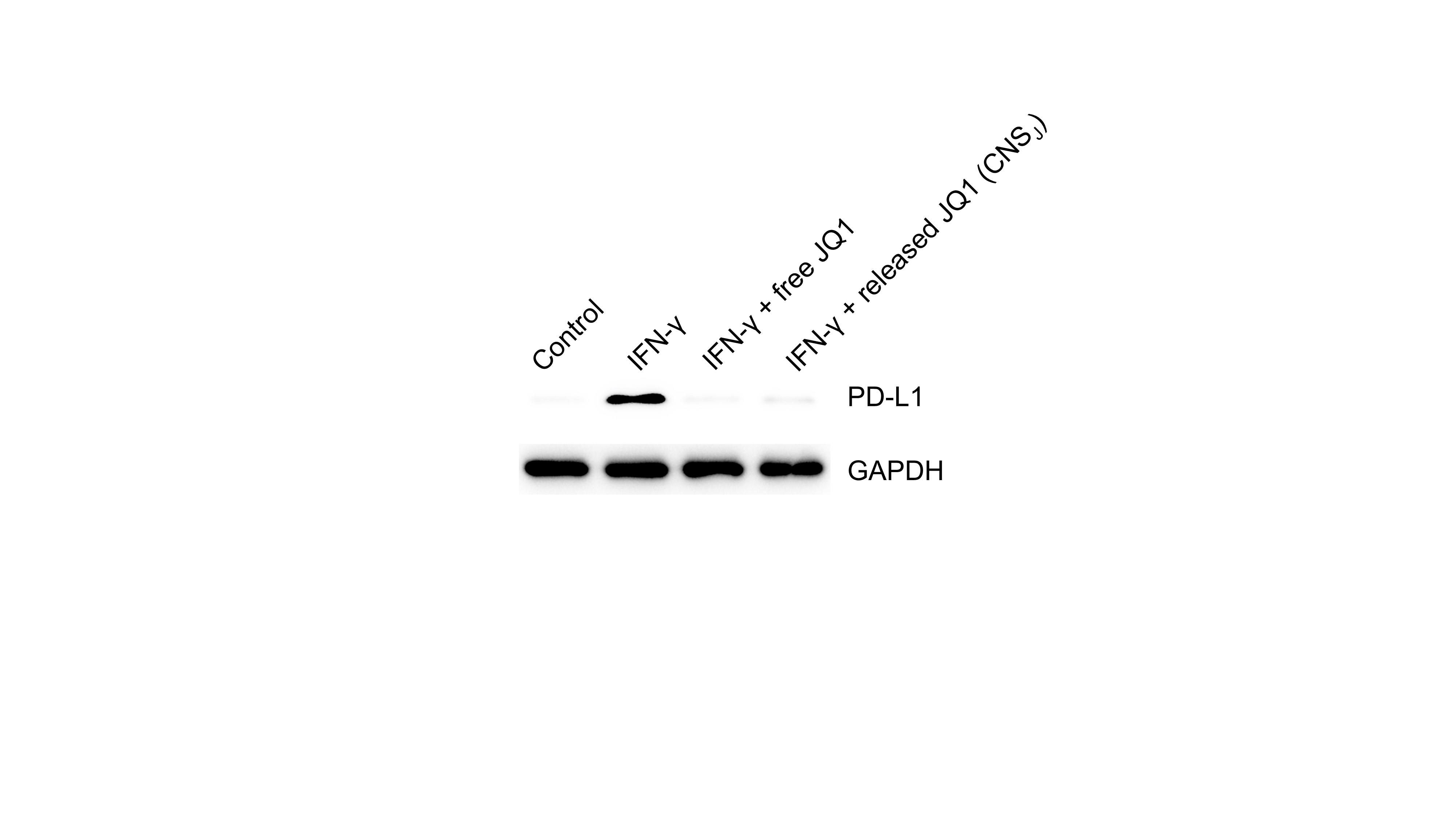


**Figure S12.** Western-blot analysis of JQ1-induced downregulation of PD-L1 in Panc02 cells (200 nM of JQ1 and 100 ng/ml of IFN-γ).


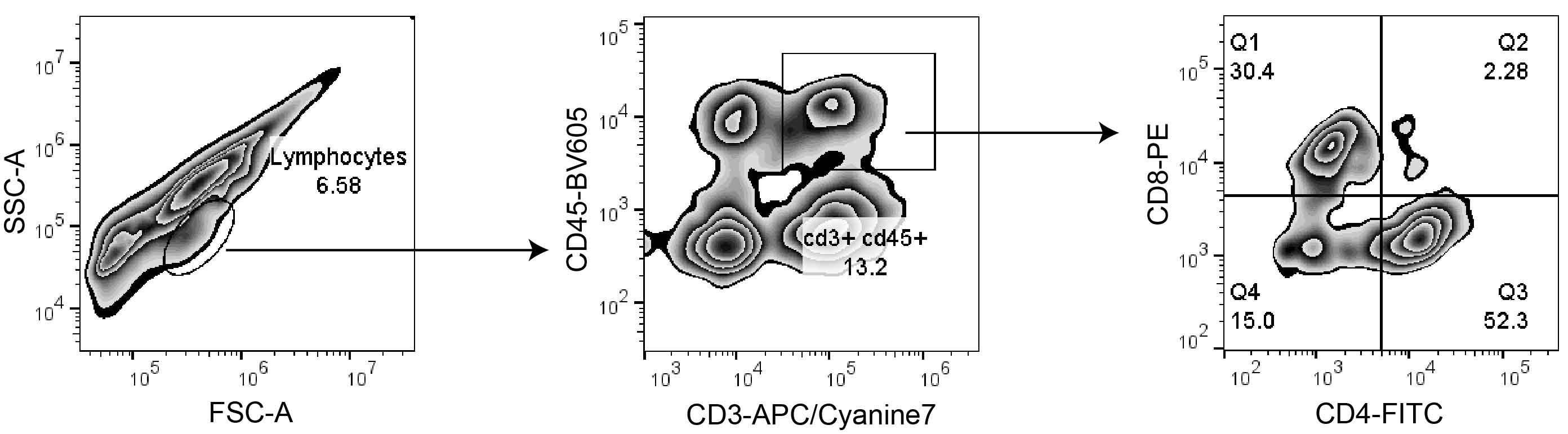


**Figure S13.** Gating strategy for flow cytometry assay of CD4^+^ T cells and CD8^+^ T cells in distant tumors of Panc02 tumor-bearing C57BL/6 mice.


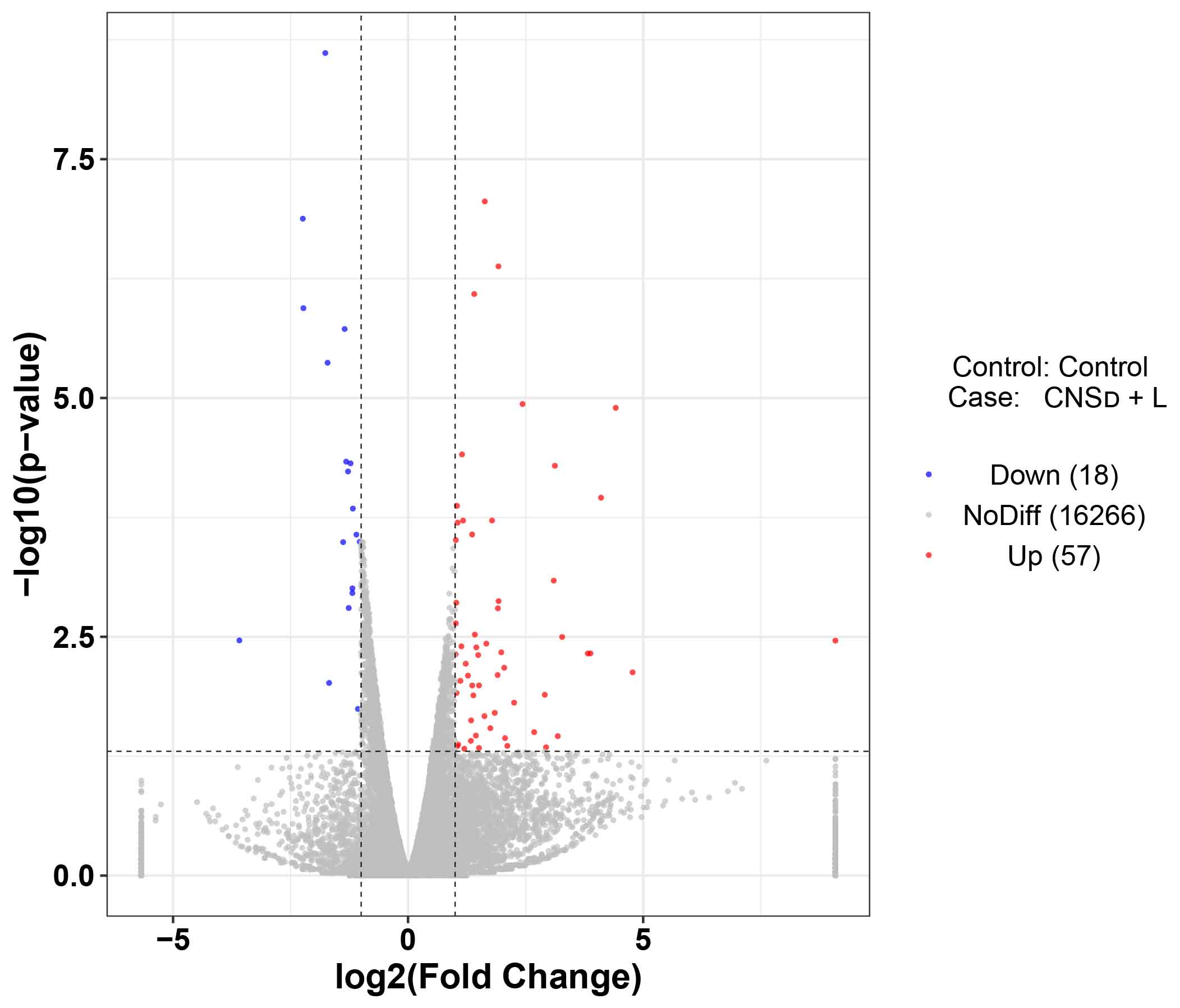


**Figure S14.** A volcano plot showing the up-regulated or insignificantly expressed or down-regulated genes when comparing the CNS_D_-treated group with the Control (PBS) group.


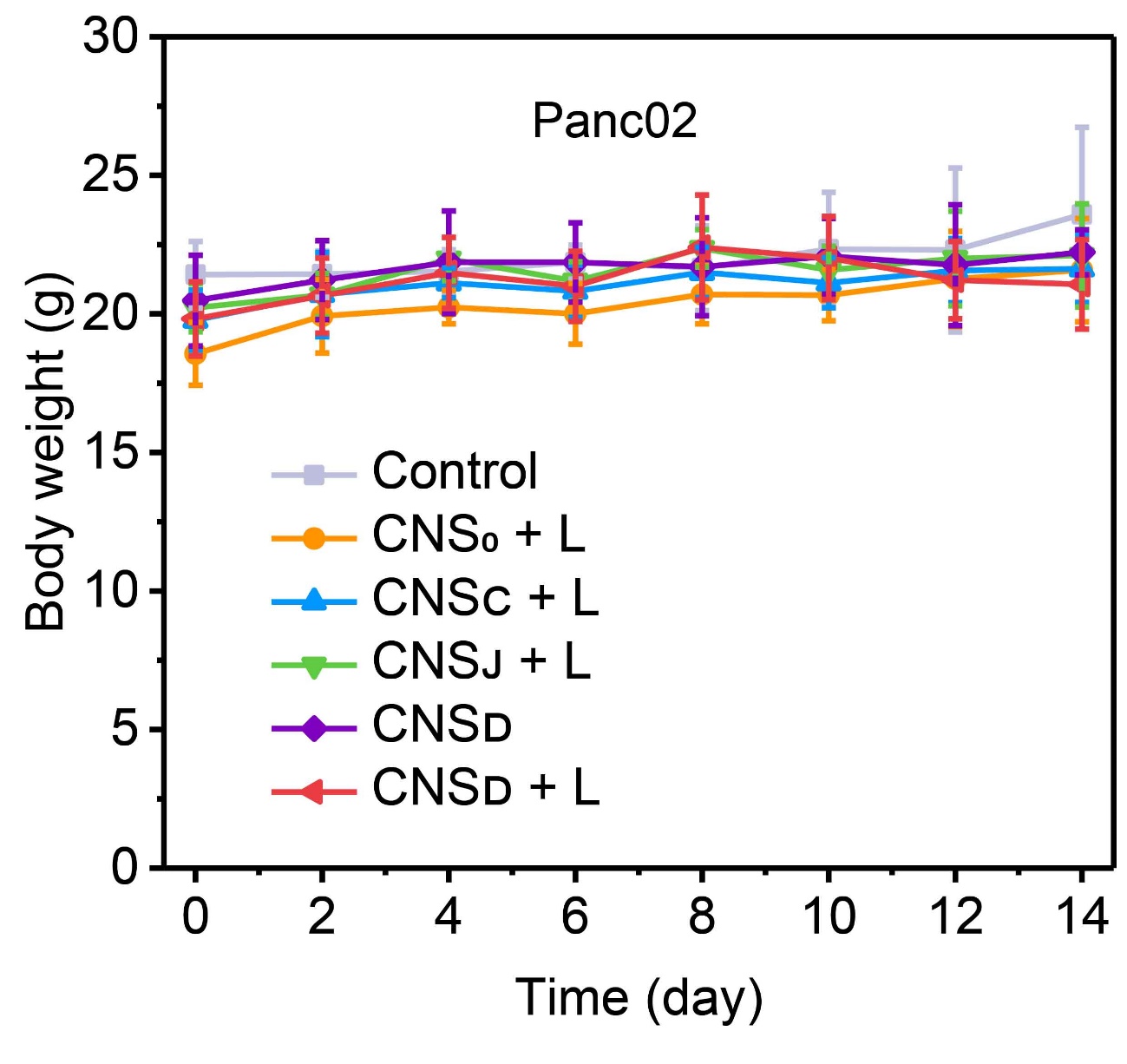


**Figure S15.** Body weights of Panc02 tumor-bearing C57BL/6 mice in different groups within 14 days of treatment (Control (PBS), CNS_0_, CNS_C_, CNS_J_ and CNS_D_, 0.2 mL, [CuS] = 300 μg/mL, n = 5).


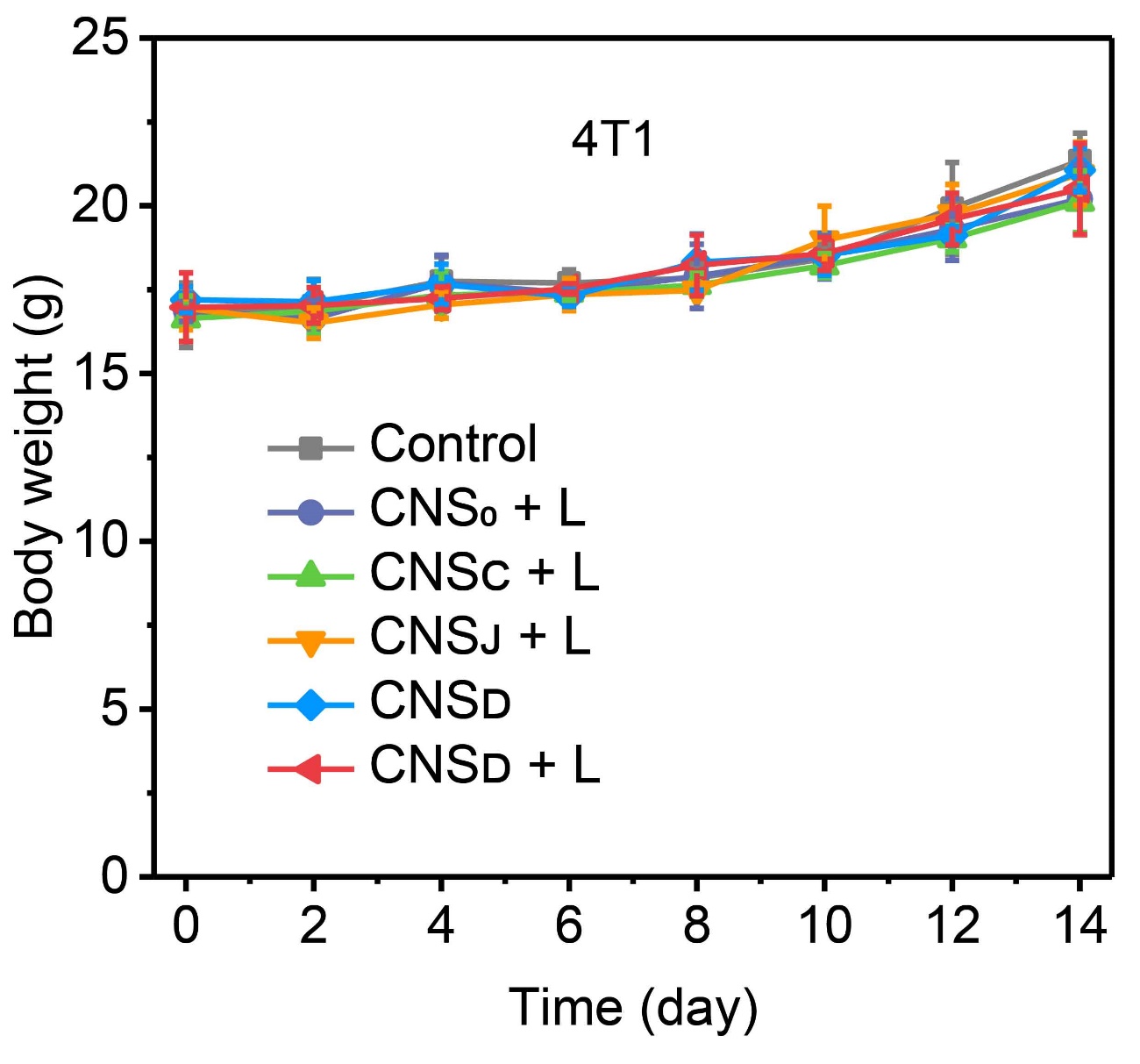


**Figure S16.** Body weights of 4T1 tumor-bearing Balb/c mice in different groups within 14 days of treatment (Control (PBS), CNS_0_, CNS_C_, CNS_J_ and CNS_D_, 0.2 mL, [CuS] = 300 μg/mL, n = 5).


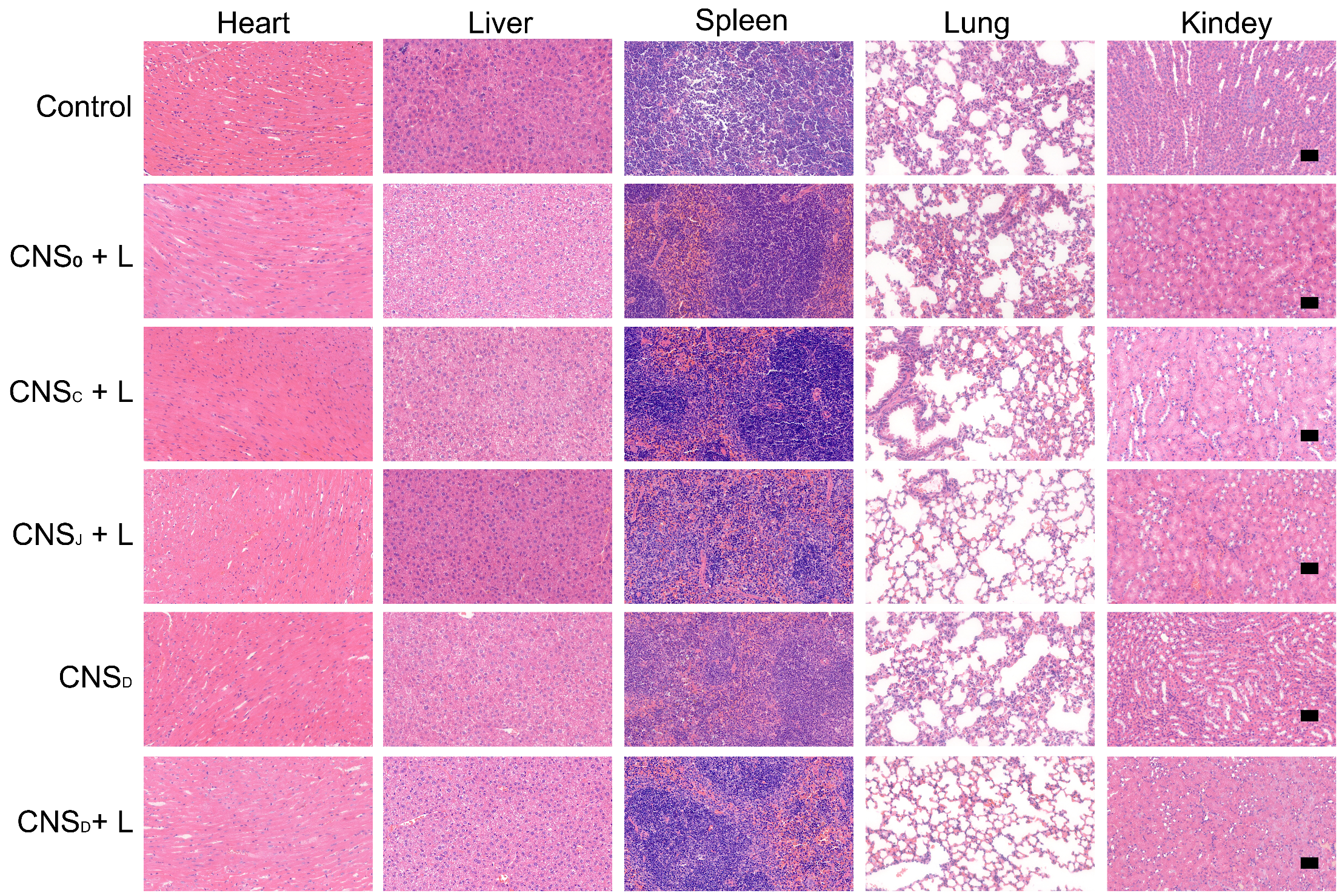


**Figure S17.** Representative histological H&E staining images of major organs (heart, liver, spleen, lung, and kidney) were collected from Panc02 tumor-bearing C57BL/6 mice in different treatment groups at the end of treatment (Control (PBS), CNS_0_, CNS_C_, CNS_J_ and CNS_D_, 0.2 mL, [CuS] = 300 μg/mL). The scale bar represents 50 μm.


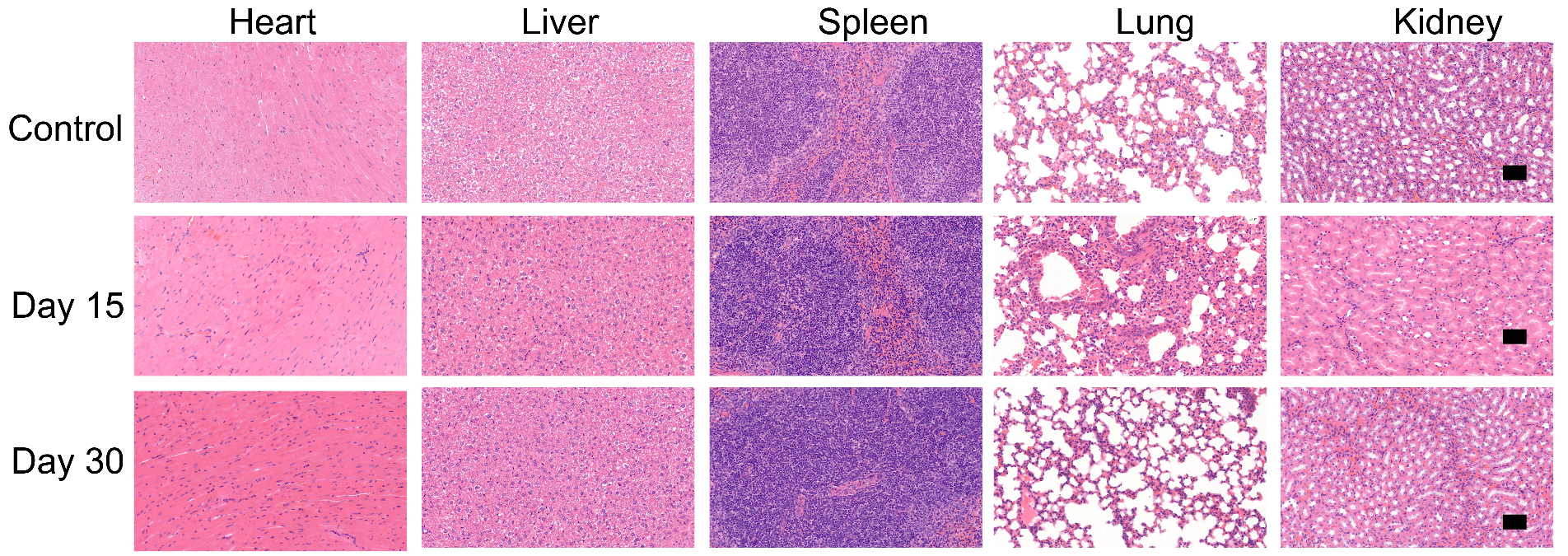


**Figure S18.** Representative histological H&E staining photos of major organs (heart, liver, spleen, lung, and kidney) in healthy C57BL/6 mice before treatment (Control) and after tail-intravenous injection of CNS_D_ (0.2 mL, [CuS] = 00 μg/mL) for 15 (Day 15) and 30 days (Day 30). The scale bar represents 50 μm.


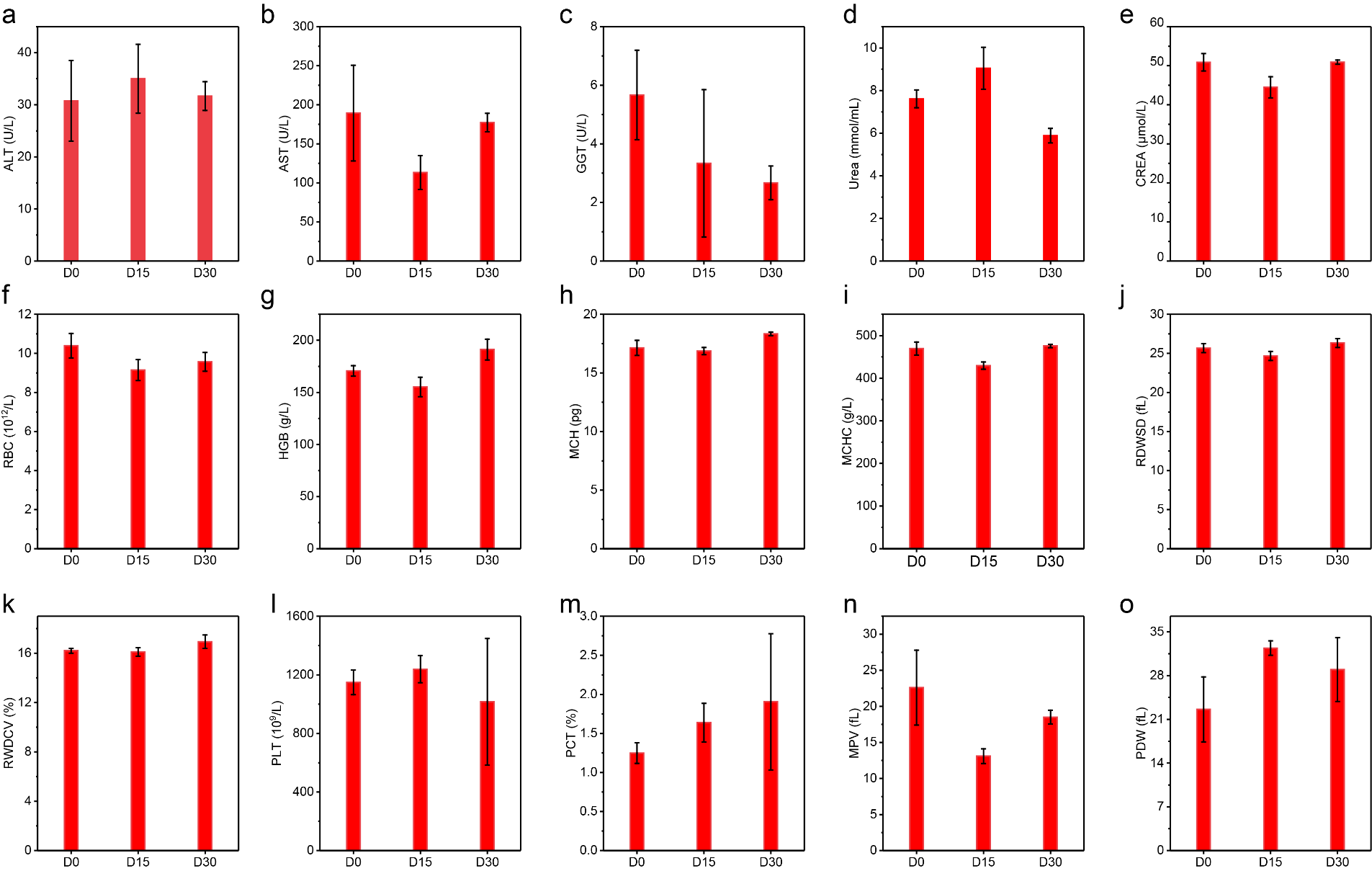


**Figure S19.** The levels of (a) alanine aminotransferase (ALT), (b) aspartate aminotransferase (AST), (c) γ-glutamyl transpeptidase (GGT), (d) urea, (e) creatinine (CREA), (f) red blood cells (RBC), (g) hemoglobin (HGB), (h) mean corpuscular hemoglobin (MCH), (i) hemoglobin concentration (MCHC), (j) red cell distribution width (RDW-SD), (k) red cell volume distribution width (RDW-CV), (l) platelet (PLT), (m) plateletcrit (PCT), (n) mean platelet volume (MPV), and (o) platelet distribution width (PDW) in the blood of healthy C57BL/6 mice before treatment (D0) and after tail-intravenous administration of CNS_D_ (0.2 mL, [CuS] = 300 μg/mL) for 15 (D15), and 30 days (D30) (n = 3).
